# Adaptive fuzzy super-twisting sliding mode control with nonlinear active disturbance rejection for electromechanical stabilizer bars to improve vehicle roll stability

**DOI:** 10.1371/journal.pone.0356645

**Published:** 2026-09-28

**Authors:** Tuan Anh Nguyen

**Affiliations:** Thuyloi University, Hanoi, Vietnam; National University of Singapore, SINGAPORE

## Abstract

Electromechanical stabilizer bars are commonly equipped in luxury vehicles to enhance rollover stability during aggressive steering maneuvers at high speeds. However, most existing studies simplify actuator and vehicle dynamics, while parametric uncertainties, model nonlinearities, and external disturbances are often insufficiently addressed. This paper proposes a robust adaptive control framework for an active anti-roll system with three main contributions. First, a comprehensive coupled dynamic model is established to explicitly capture the interaction between the electromechanical stabilizer-bar actuator and vehicle dynamics. Second, an integrated Super-Twisting Sliding Mode Control and Nonlinear Active Disturbance Rejection Control scheme is developed, in which the nonlinear extended state observer estimates and compensates for lumped disturbances and uncertainties, while the super-twisting mechanism provides robust motor current tracking. Third, an adaptive fuzzy gain-scheduling mechanism adjusts the control gain online according to vehicle operating conditions. The novelty therefore lies in the coordinated integration of coupled actuator–vehicle modeling, disturbance compensation, robust current tracking, and operating-condition-dependent gain adaptation within an electromechanical stabilizer-bar control framework. Numerical simulations demonstrate superior performance to the benchmark controllers in reducing vertical tire-force variations and improving vehicle dynamics. Under a J-turn maneuver at 100 km/h, the maximum motor-current tracking error is limited to 0.11 A, with an overshoot of 3.59% and a settling time of 0.17 s. These results demonstrate the effectiveness and potential of the proposed framework for automotive active control systems.

## 1 Introduction

Vehicle rollover accidents occur relatively infrequently; however, they often result in severe consequences, particularly for heavy-duty vehicles [1]. The causes of rollover are multifaceted, involving driving conditions, vehicle geometric parameters, and steering maneuvers [2]. Alrejjal et al. [3] identified that weather conditions, road characteristics, and vehicle speed have a significant influence on rollover instability. Hassan et al. [4] emphasized that tire–road adhesion and suspension system parameters play a crucial role in maintaining rollover stability during steering. A study conducted by Alrejjal and Ksaibati further demonstrated that crosswinds have a considerable impact on vehicle rollover behavior [5]. Xu et al. [6] reported that steering maneuvers are a key factor in ensuring vehicle stability during high-speed cornering. Similarly, Xin et al. [7] confirmed that aggressive steering at high speeds substantially increases the risk of rollover. Moreover, Tan et al. [8] verified that vehicles with a high center of gravity, such as Sport Utility Vehicles (SUVs), are more prone to rollover under high-speed steering conditions.

In recent years, various controllable active safety systems have been introduced in modern vehicles to enhance stability during steering. Chen et al. [9] investigated several advanced anti-roll strategies aimed at improving vehicle roll dynamics. Tan et al. [10] reported that uneven load distribution can significantly increase the risk of vehicle rollover and subsequently proposed advanced control solutions for motor-in-wheel systems to mitigate this issue. Arslan and Sever [11] designed a constrained Model Predictive Control (MPC) scheme based on quadratic programming to control an active steering system, thereby improving yaw rate response and vertical load transfer. A rollover prevention strategy based on steer-by-wire control using MPC with a barrier cost function was presented by Ataei et al. in [12]. Yao et al. [13] proposed an H∞ control approach for active suspension systems to compensate rollover-induced moments, and simulation results demonstrated a significant reduction in roll angle compared with passive suspension systems. Qian et al. [14] developed an integrated control framework combining MPC (to reduce steering angle) and Sliding Mode Control (SMC) (to redistribute yaw moment during braking) based on a simplified vehicle dynamic model, to enhance rollover stability. Furthermore, Wu et al. [15] developed a Nash game-based control strategy grounded in Linear Quadratic Regulator (LQR) theory to coordinate steering and braking systems for improved rollover stability. Zhou et al. [16] introduced a Kalman Filter (KF) integrated with a sliding-mode observer to estimate roll angle variations and designed a hierarchical MPC scheme with time-varying weights and constraints to enhance the control effectiveness of engine torque, differential braking, and active steering. Although the aforementioned approaches can improve rollover stability under certain operating conditions, they are often associated with high implementation costs, and rollover risks cannot be eliminated.

Equipping vehicles with stabilizer bars is an effective solution for enhancing rollover resistance. Nguyen et al. [17] introduced two types of active stabilizer bars: hydraulic stabilizer bars and electromechanical stabilizer bars, both of which exhibit superior performance compared to conventional passive stabilizer bars. Muniandy et al. [18] reported that active stabilizer bars are considerably more cost-effective than fully active suspension systems while still providing substantial performance improvements. In recent years, several studies have focused on the control of hydraulic stabilizer bars. Huang et al. [19] presented a virtual functional testing framework for the hydraulic actuator of an active stabilizer bar. Although the dynamics of the hydraulic valve system were considered in their study, the structure of the control strategy was not explicitly addressed. An application of Proportional–Integral–Derivative (PID) control for controlling the hydraulic valve system within a stabilizer bar actuator was introduced in [20]. However, the proposed control scheme is relatively simple, lacks optimal design considerations, and does not rigorously address system stability. Dawei et al. [21] conducted experimental validation of a fuzzy PID controller designed for hydraulic stabilizer bar control. Nevertheless, the proposed fuzzy algorithm employs fixed membership functions and rule bases, resulting in limited adaptability. Nemeth and Varga [22] proposed a hierarchical Linear Parameter-Varying (LPV) control approach to regulate the performance of a hydraulic stabilizer bar under actuator current saturation. In addition, several fuzzy control strategies have recently been developed to enhance rollover stability during high-speed steering by considering both road holding and ride comfort [23,24]. Despite their effectiveness under certain conditions, these approaches often suffer from performance degradation due to non-optimized or non-adaptive control structures.

The structure of hydraulic stabilizer bar systems is relatively bulky and complex, and the influence of time delay remains unavoidable. To overcome these limitations, electromechanical stabilizer bars have been increasingly adopted in high-end vehicles as an alternative to hydraulic stabilizer bars. A representative configuration of an electromechanical stabilizer bar system implemented in the Audi AQ7 was introduced by Sagewka et al. [25]. Buma et al. [26] developed an electrical actuator model capable of delivering a maximum torque of up to 665 Nm; however, the corresponding control system architecture was not fully described, which limits its reproducibility and practical accessibility. Kong et al. [27] proposed a hierarchical control approach consisting of an SMC algorithm for computing the target active anti-roll torque at the upper layer, a fuzzy logic algorithm for torque distribution, and a PI controller for regulating the actuator output torque. Dawei et al. [28] developed a coordinated control strategy for integrated active steering and electromechanical stabilizer bar systems based on fuzzy PID and SMC techniques. Nevertheless, the proposed fuzzy control structure is relatively simple and lacks adaptability, while the actuator dynamics are not discussed in sufficient detail. Anafjeh et al. [29] compared the performance of three controllers, including Linear Quadratic Gaussian (LQG), PI, and MPC combined with a KF, for controlling an electromechanical stabilizer bar system applied to railway vehicles. Simulation results indicated that the PI controller produced unstable lateral acceleration responses, whereas the LQG controller suffered from excessive overshoot. More recently, Nguyen [30] proposed an SMC-based approach with a sliding surface constructed using an expanded sigmoid function tuned by a dynamic fuzzy mechanism. Although the output of the fuzzy algorithm was optimized through a scaling gain based on a performance index function, its adaptability was still not rigorously guaranteed.

Several extended applications of SMC have recently been developed to enhance vehicle rollover stability. Liu et al. [31] proposed a fixed-time adaptive SMC approach based on an improved super-twisting observer to enhance yaw and roll stability. Huang et al. [32] introduced an adaptive SMC scheme that incorporates adjustable barrier functions to enhance vehicle rollover stability during steering. Another application of Super-Twisting Sliding Mode Control (STSMC) for active steering systems was reported by Chen et al. [33]. In addition, several hybrid control frameworks combining Active Disturbance Rejection Control (ADRC) with nonlinear MPC [34], as well as ADRC integrated with SMC [35,36], have demonstrated high effectiveness in controlling electromechanical systems subjected to disturbances and uncertainties.

Some similarity exists in the mathematical modeling of vehicle steering dynamics because the same fundamental vehicle dynamics are required to describe steering maneuvers. However, the control objectives and methodologies are substantially different. Nguyen [37] developed a fuzzy control strategy for a hydraulic stabilizer bar, demonstrating the effectiveness of fuzzy control in improving vehicle rollover stability. More recently [38], proposed a nonlinear ADRC-based sliding-mode controller for an Electric Power Assisted Steering (EPAS) system to enhance steering performance by estimating disturbances. Although these studies provide useful insights, they focus on different actuators, mathematical models, and control objectives. In contrast, the present work addresses the control of an electromechanical stabilizer bar by explicitly considering the electromechanical actuator dynamics and their coupling with the full vehicle dynamics. Furthermore, an Adaptive Fuzzy STSMC integrated with Nonlinear ADRC is developed to improve robustness, disturbance rejection, and adaptability under uncertain operating conditions.

Recent studies have also investigated coordinated chassis control for improving the dynamic performance of electric vehicles. In [39], a decentralized multi-agent framework integrating active front steering and active suspension was developed using distributed model predictive control, achieving improved lateral and vertical stability while considering ride comfort and roll safety. More recently [40], integrated torque vectoring and active suspension for distributed-drive electric vehicles using a multi-agent framework that combines distributed model predictive control and game theory, demonstrating improved vehicle dynamic performance while maintaining ride comfort. These studies demonstrate the potential of coordinated chassis-control systems; however, robust roll control using electromechanical stabilizer bars, while explicitly accounting for actuator electromechanical dynamics and uncertainties, remains comparatively underexplored.

Recent developments in super-twisting-based control have demonstrated its effectiveness in addressing robustness and chattering problems across nonlinear and uncertain systems. Rsetam et al. [41] applied super-twisting integral SMC to a flexible-joint robot, combining the robustness of integral sliding mode with the continuous super-twisting action to improve trajectory tracking and reduce chattering. More recently, a model-based smooth STSMC was developed for nonlinear cancer chemotherapy models subject to parametric uncertainties and external disturbances, achieving smoother control action and improved disturbance rejection compared with conventional STSMC [42]. For permanent magnet synchronous motors, Zheng et al. [43] integrated super-twisting integral terminal SMC with an extreme learning machine, where the latter compensates for lumped disturbances and relaxes the disturbance-bound requirement of the controller. These studies illustrate the evolution of super-twisting techniques toward smoother control, faster convergence, and enhanced disturbance compensation in nonlinear systems.

Observer-based disturbance rejection has also evolved toward greater robustness to increasingly complex uncertainties. Rsetam et al. [44] combined a cascaded ESO with SMC for an underactuated flexible-joint robot, using the observer to estimate unmeasured states and lumped disturbances while reducing noise amplification and peaking effects. Subsequently, an adaptive ESO with error-dependent observer gains was incorporated into the ADRC for an electric furnace to improve disturbance estimation and alleviate high-gain and noise-sensitivity problems [45]. This adaptive observer concept was further applied to a nonlinear steer-by-wire system with communication delays, in which the observer estimates unmeasured states and compound disturbances, including parameter uncertainties, nonlinearities, external disturbances, and delay effects [46]. More recently, a generalized proportional-integral observer was integrated with continuous SMC for networked servo systems to estimate unmatched disturbances arising from communication delays, and its feasibility was further demonstrated experimentally [47]. These developments show a clear progression from state and disturbance estimation toward adaptive and delay-aware observer structures integrated with robust control.

Recent studies have brought these robust-control and disturbance-rejection concepts closer to electromechanical stabilizer bar applications. A detailed coupled vehicle–actuator model with Fixed-time SMC (FxTSMC) was developed in [48], demonstrating the benefit of explicitly accounting for electric anti-roll-bar dynamics rather than assuming ideal force generation. A disturbance-rejection-enhanced smooth SMC framework was subsequently proposed in [49], where an LESO and a fuzzy-tuned sigmoid switching function were employed to improve disturbance rejection, control smoothness, and adaptability. In [50], nonlinear ADRC was integrated with fuzzy-enhanced finite-time SMC, using an NESO to estimate uncertainties and external disturbances while adaptively adjusting the switching gain. More recently, FxTSMC and ADRC were combined within a comprehensive nonlinear electromechanical stabilizer bar model to exploit the complementary advantages of rapid convergence and active disturbance compensation [51]. Collectively, these studies indicate a transition from standalone robust control toward integrated observer-based and adaptive control architectures. Nevertheless, the coordinated integration of nonlinear disturbance estimation, super-twisting robust tracking, and online adaptive gain scheduling within a coupled electric stabilizer bar–vehicle model remains insufficiently investigated.

### 1.1 Research gaps and key contributions

Overall, research on the control of electromechanical stabilizer bar systems remains relatively limited. Although notable progress has been achieved in rollover prevention through the application of electromechanical stabilizer bars, several critical issues remain unresolved. First, many existing studies neglect the coupling effects of vehicle dynamics, while the actuator dynamics are often described in an oversimplified manner. Second, the influence of adverse factors, including model nonlinearities, external disturbances, and parametric uncertainties, has not been sufficiently addressed during system modeling and control design. Third, commonly used control strategies, such as PID, LQR, and ADRC, still suffer from overshoot issues, whereas SMC–based approaches are prone to chattering phenomena. Finally, the mathematical structure of fuzzy-based tuning mechanisms is typically fixed, resulting in limited adaptability under varying operating conditions. These limitations constitute the remaining research gaps in the current literature.

These research gaps motivate the development of a control framework that can simultaneously account for the coupled vehicle–actuator dynamics, compensate for adverse effects, and maintain robust and adaptive performance under varying operating conditions. Accordingly, this paper proposes a robust control framework that integrates STSMC and Nonlinear ADRC (NADRC). The effects of adverse factors are estimated using a Nonlinear Extended State Observer (NESO) and compensated through the NADRC-based control law. At the same time, STSMC is employed to ensure robust tracking performance and mitigate overshoot and chattering. Additionally, the control parameters are adaptively updated via an adaptive fuzzy gain-scheduling mechanism, thereby enhancing system adaptability under varying operating conditions. Moreover, the interaction between vehicle and actuator dynamics is considered comprehensively, explicitly accounting for adverse effects. These aspects constitute the main contributions of this paper and distinguish the proposed approach from existing studies.

The remainder of this paper is organized as follows. The background review, research gaps, and key contributions are presented in Section 1. Section 2 develops the dynamic model of the system. The integrated control strategy is designed in Section 3. Simulation results and discussions are provided in Section 4. Finally, Section 5 concludes the paper and outlines potential directions for future research.

## 2 Dynamic models

A spatial dynamic model with seven degrees of freedom is illustrated in Fig 1. By applying the multi-body system decomposition principle, the vehicle is divided into one sprung mass (*m*_*s*_) and four unsprung masses (*m*_*u*_).

**Fig 1:**
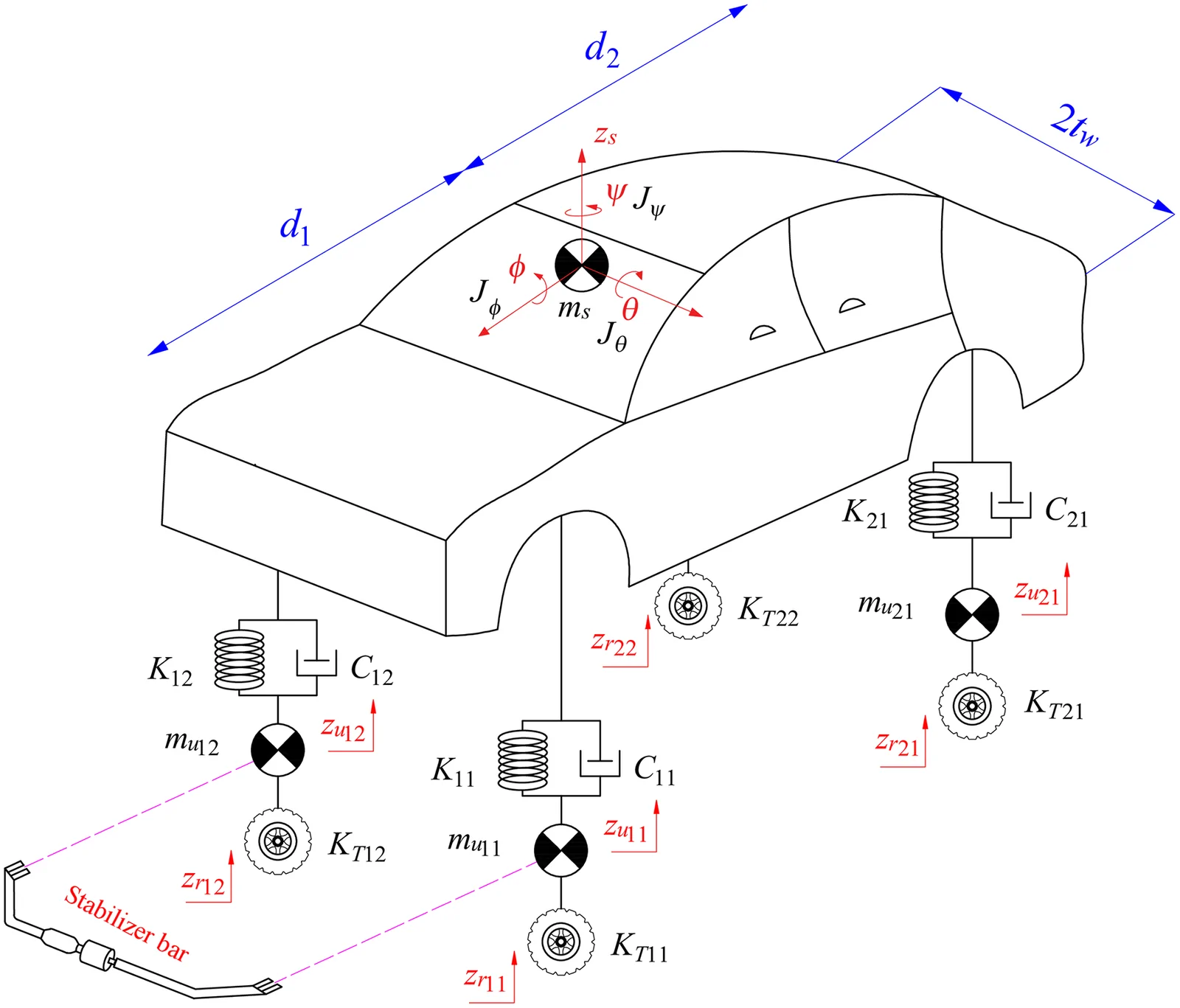
Vehicle dynamics model.

The system dynamics are described by equations (1)–(4) based on the Newton–Euler formulation [30], where *z*_*s*_ is sprung mass displacement, *z*_*u*_ is unsprung mass displacement, *J*_*ϕ*_ is roll inertia, *ϕ* is roll angle, *h*_*ϕ*_ is roll center height, *J*_*θ*_ is pitch inertia, *θ* is pitch angle, *h*_*θ*_ is pitch center height, 2*t*_*w*_ is trackwidth, *d* is axle distance, *g* is gravitational acceleration, and *F*_*A*_ is actuator force.


$$\begin{array}{c} \sum_{i,j=1}^{\text{2}}\left(F_{Kij}+F_{Cij}\right)=m_{s}{\ddot{z}}_{s} \end{array}$$ (1)

$$\begin{array}{c} \sum_{i,j=1}^{\text{2}}\left[{\left(-1\right)}^{j-1}\left(F_{Kij}+F_{Cij}\right)t_{wi}\right]+\left(a_{y}cos\phi +gsin\phi \right)m_{s}h_{\phi }=\left(J_{\phi }+m_{s}h_{\phi }^{\text{2}}\right)\ddot{\phi } \end{array}$$ (2)

$$\begin{array}{c} \sum_{i,j=1}^{\text{2}}{\left(-1\right)}^{i-1}\left(F_{Kij}+F_{Cij}\right)d_{i}=\left(J_{\theta }+m_{s}h_{\theta }^{\text{2}}\right)\ddot{\theta } \end{array}$$ (3)

$$\begin{array}{c} F_{Tij}-F_{Kij}-F_{Cij}+{\left(-1\right)}^{j}F_{A}=m_{uij}{\ddot{z}}_{uij} \end{array}$$ (4)

The lateral acceleration (*a*_*y*_) depends on the heading angle (*β*) and the yaw angle (*ψ*), as expressed in (5). The heading angle is defined based on the relationship between the lateral velocity (*v*_*y*_) and the longitudinal velocity (*v*_*x*_), as given in (6).


$$\begin{array}{c} a_{y}=\left(\dot{\beta }+\dot{\psi }\right)v_{x}+{\dot{v}}_{y} \end{array}$$ (5)

$$\begin{array}{c} tan\beta =\frac{v_{y}}{v_{x}} \end{array}$$ (6)

The suspension interaction forces consist of the spring force (*F*_*K*_) and the damping force (*F*_*C*_). They are calculated according to (7) and (8), respectively. As given in (9), the tire spring force (*F*_*T*_) depends on the unsprung mass displacement and the road excitation (*z*_*r*_). The symbols used are as follows: *K* denotes the suspension spring coefficient, *C* represents the damping coefficient, and *K*_*T*_ is the tire spring coefficient.


$$\begin{array}{c} F_{Kij}=K_{ij}\left[z_{s}-z_{uij}+{\left(-1\right)}^{j+1}t_{wi}\phi +{\left(-1\right)}^{i+1}d_{i}\theta \right] \end{array}$$ (7)

$$\begin{array}{c} F_{Cij}=C_{ij}\left[{\dot{z}}_{s}-{\dot{z}}_{uij}+{\left(-1\right)}^{j+1}t_{wi}\dot{\phi }+{\left(-1\right)}^{i+1}d_{i}\dot{\theta }\right] \end{array}$$ (8)

$$\begin{array}{c} F_{Tij}=K_{Tij}\left(z_{rij}-z_{uij}\right) \end{array}$$ (9)

A nonlinear motion model (Fig 2) is established using equations (10)–(12) to describe the evolution of the yaw angle, where *δ* denotes the steering angle, *J*_*ψ*_ is the yaw moment of inertia, *M*_*z*_ represents the self-aligning moment, *F*_*x*_ is the longitudinal force, and *F*_*y*_ is the lateral force. The interaction between the tire and the road surface is modeled using a nonlinear Pacejka tire formulation.


$$\begin{array}{c} \sum_{i,j=1}^{\text{2}}\left(F_{xij}cos\delta_{ij}-F_{yij}sin\delta_{ij}\right)=\left[{\dot{v}}_{x}-\left(\dot{\beta }+\dot{\psi }\right)v_{y}\right]\left(m_{s}+\sum_{i,j=1}^{\text{2}}m_{uij}\right) \end{array}$$ (10)

$$\begin{array}{c} \sum_{i,j=1}^{\text{2}}\left(F_{xij}sin\delta_{ij}+F_{yij}cos\delta_{ij}\right)=\left[{\dot{v}}_{y}+\left(\dot{\beta }+\dot{\psi }\right)v_{x}\right]\left(m_{s}+\sum_{i,j=1}^{\text{2}}m_{uij}\right) \end{array}$$ (11)

$$\begin{array}{c} \sum_{i,j=1}^{\text{2}}\left[{\left(-1\right)}^{j}\left(F_{xij}cos\delta_{ij}-F_{yij}sin\delta_{ij}\right)t_{wi}+{\left(-1\right)}^{i+1}\left(F_{xij}sin\delta_{ij}+F_{yij}cos\delta_{ij}\right)d_{i}-M_{zij}\right]=J_{\psi }\ddot{\psi } \end{array}$$ (12)

**Fig 2:**
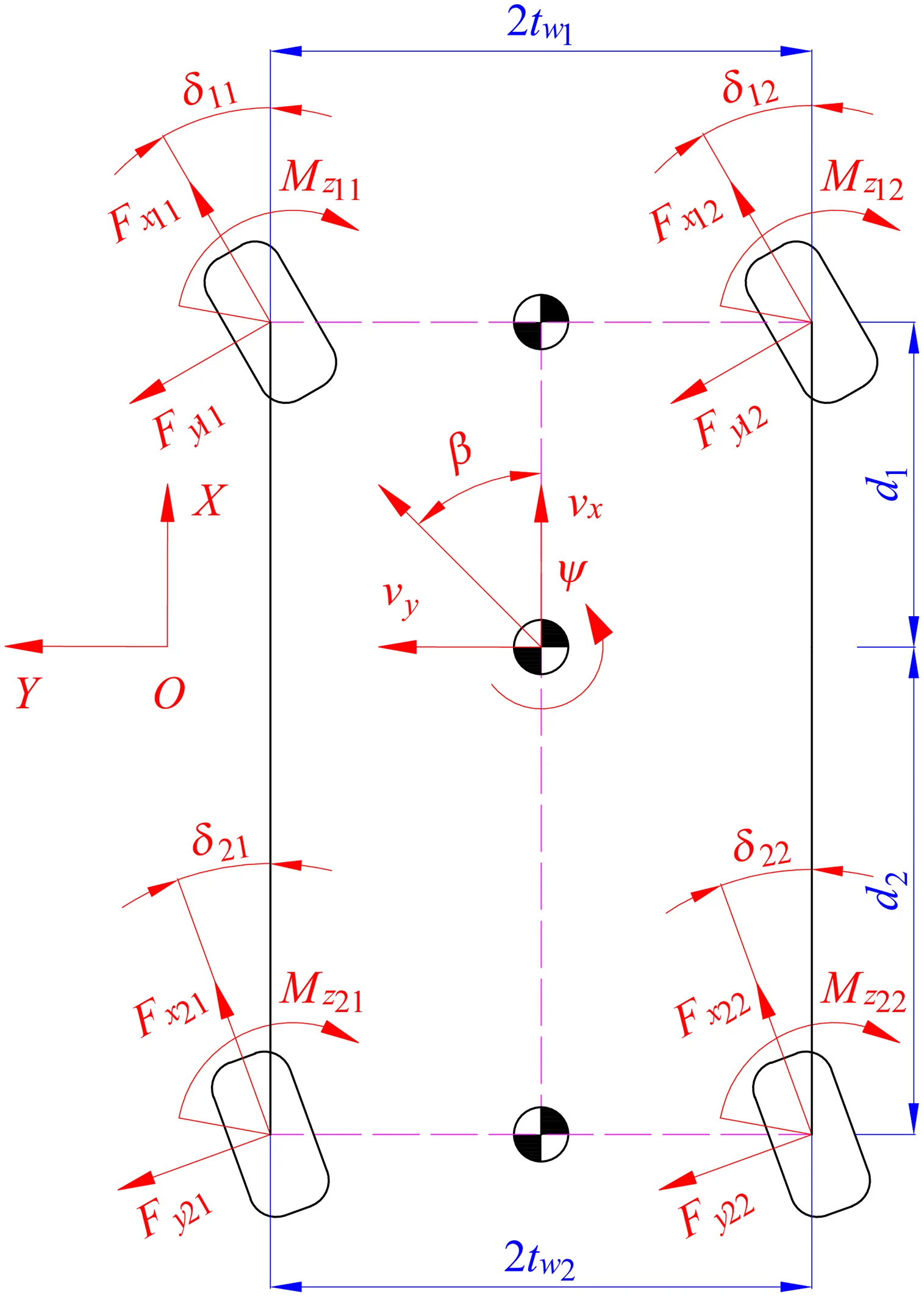
Double-track dynamics model.

The actuator force (*F*_*A*_), generated by the electromechanical stabilizer bar, is defined by equation (13), where *l*_*b*_ denotes the bar arm length, *T*_*c*_ is the bar torque, *c*_*c*_ represents the coupling joint damping coefficient, and *k*_*c*_ is the coupling joint stiffness.


$$\begin{array}{c} F_{A}=\frac{T_{c}}{l_{b}}=\frac{c_{c}\left({\dot{\theta }}_{o}-{\dot{\theta }}_{b}\right)+k_{c}\left(\theta_{o}-\theta_{b}\right)}{l_{b}} \end{array}$$ (13)

The actuator dynamics are described by equations (14)–(17), where *θ*_*m*_ denotes the motor shaft position, *θ*_*o*_ is the output shaft position, *θ*_*b*_ represents the stabilizer bar position, and *i*_*g*_ is the motor gear ratio. In addition, *J*_*o*_ and *J*_*b*_ denote the output shaft inertia and the bar inertia, respectively, while *c*_*o*_ and *c*_*b*_ are the output shaft and bar damping coefficients, and *k*_*b*_ is the bar stiffness. Furthermore, *i*_*m*_ represents the motor current, *R*_*m*_ is the motor resistance, *L*_*m*_ is the motor inductance, *k*_*e*_ denotes the back electromotive force coefficient, *η* is the transmission efficiency, *k*_*t*_ is the torque constant, and *u* is the control input.


$$\begin{array}{c} \theta_{m}=i_{g}\theta_{o} \end{array}$$ (14)

$$\begin{array}{c} i_{g}i_{m}k_{t}\eta =J_{o}{\ddot{\theta }}_{o}+c_{o}{\dot{\theta }}_{o}+T_{c} \end{array}$$ (15)

$$\begin{array}{c} T_{c}=J_{b}{\ddot{\theta }}_{b}+c_{b}{\dot{\theta }}_{b}+k_{b}\theta_{b} \end{array}$$ (16)

$$\begin{array}{c} u=L_{m}{\dot{i}}_{m}+R_{m}i_{m}+k_{e}{\dot{\theta }}_{m} \end{array}$$ (17)

Substituting equations (13) and (14) into equations (15) and (16) yields equations (18) and (19). External disturbances (*T*_*d*_), model nonlinearities (*R*_*n*_), and parametric uncertainties (Δ*R*_*m*_, Δ*L*_*m*_, and Δ*k*_*e*_) are taken into account in this work. Accordingly, equation (17) is rewritten in a more detailed form, as given in equation (20).


$$\begin{array}{c} i_{g}i_{m}k_{t}\eta =J_{o}{\ddot{\theta }}_{o}+\left(c_{o}+c_{c}\right){\dot{\theta }}_{o}+k_{c}\theta_{o}-c_{c}{\dot{\theta }}_{b}-k_{c}\theta_{b} \end{array}$$ (18)

$$\begin{array}{c} c_{c}{\dot{\theta }}_{o}+k_{c}\theta_{o}=J_{b}{\ddot{\theta }}_{b}+\left(c_{b}+c_{c}\right){\dot{\theta }}_{b}+\left(k_{b}+k_{c}\right)\theta_{b} \end{array}$$ (19)

$$\begin{array}{c} u=\left(L_{m0}+\Delta L_{m}\right){\dot{i}}_{m}+\left(R_{m0}+\Delta R_{m}+R_{n}i_{m}^{\text{2}}\right)i_{m}+\left(k_{e0}+\Delta k_{e}\right)i_{g}{\dot{\varphi }}_{o}-T_{d} \end{array}$$ (20)

The coupled vehicle–actuator formulation plays an important role in the subsequent control design. Unlike a simplified model in which the commanded anti-roll force or torque is assumed to be generated instantaneously, the actual actuator force in the present model is determined by the motor’s electrical dynamics, the transmission dynamics, and the mechanical interactions among the output shaft, stabilizer bar, and vehicle suspension. Consequently, the required anti-roll action at the vehicle level must be achieved through accurate motor current regulation while accounting for the actuator’s dynamic response and uncertainties. This coupling establishes a physical interaction between the control voltage and motor current and the generated actuator force, and ultimately the vehicle roll response. Neglecting these intermediate dynamics may lead to an overestimation of achievable tracking and roll-stabilization performance because actuator lag, electromechanical dynamics, and parameter variations are not accounted for. Therefore, the coupled model provides a more appropriate basis for designing and evaluating the proposed robust controller under non-ideal operating conditions.

## 3 Control design

A high-performance robust control framework is developed in this work to control the electromechanical anti-roll bar system. The proposed scheme integrates Adaptive Fuzzy STSMC (AFSTSMC) and NADRC, thereby exploiting the complementary advantages of these constituent control techniques. The overall control architecture is illustrated in Fig 3.

**Fig 3:**
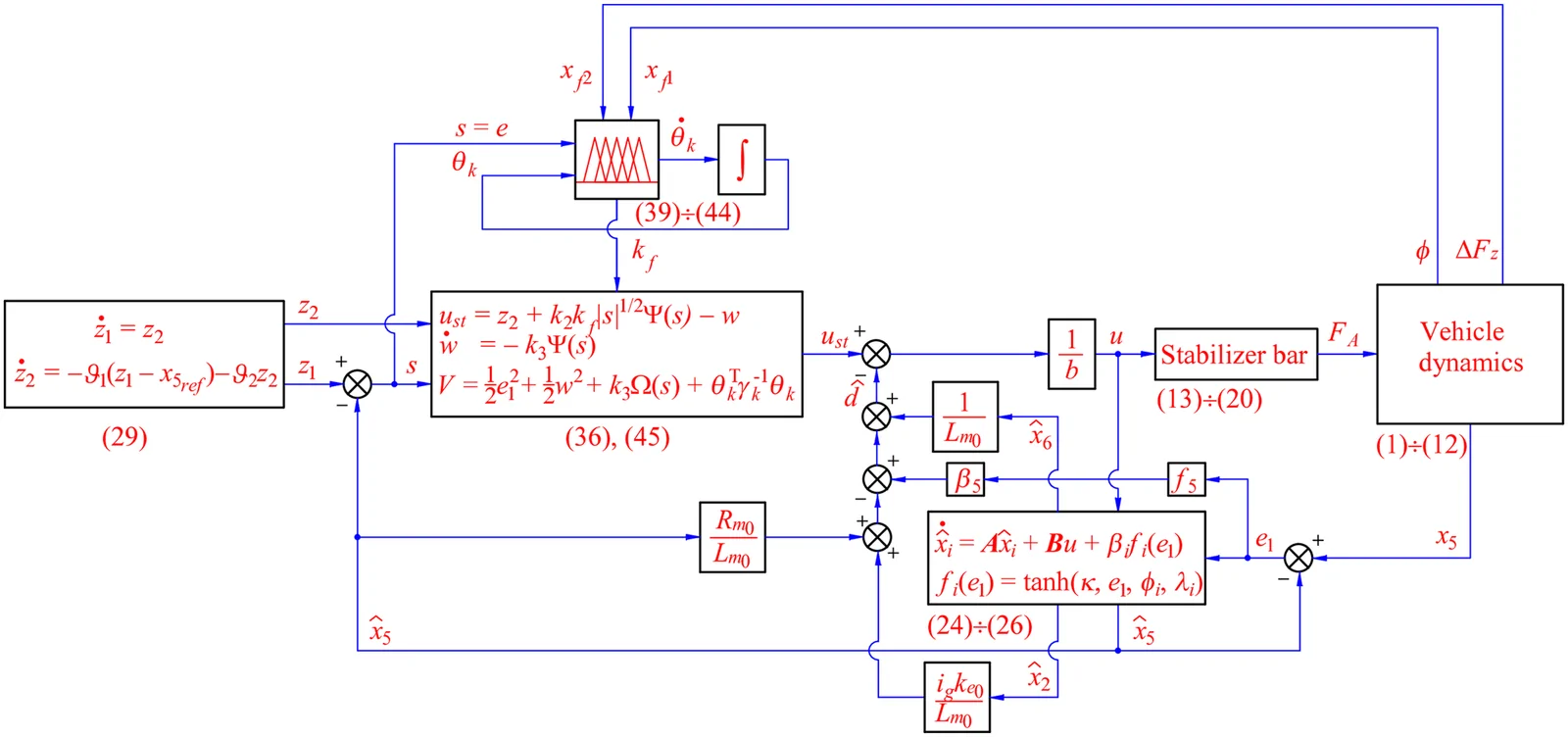
Proposed control scheme.

The state variables are arranged in the order of their appearance, as defined in equation (21).


$$\begin{array}{c} {\left[\begin{array}{ccccc} x_{\text{1}} & x_{\text{2}} & x_{\text{3}} & x_{\text{4}} & x_{\text{5}} \end{array}\right]}^{T}={\left[\begin{array}{ccccc} \theta_{o} & {\dot{\theta }}_{o} & \theta_{b} & {\dot{\theta }}_{b} & i_{m} \end{array}\right]}^{T} \end{array}$$ (21)

Equation (22) is obtained by taking the time derivatives of the state variables. The external disturbances, model nonlinearities, and parametric uncertainties are lumped into an augmented state, denoted as *x*_6_.


$$\begin{array}{c} \left\{ \begin{array}{c} {\dot{x}}_{\text{1}}=x_{\text{2}}\\ {\dot{x}}_{\text{2}}=-\frac{k_{c}}{J_{o}}x_{\text{1}}-\frac{c_{c}+c_{o}}{J_{o}}x_{\text{2}}+\frac{k_{c}}{J_{o}}x_{\text{3}}+\frac{c_{c}}{J_{o}}x_{\text{4}}+\frac{i_{g}k_{t}\eta }{J_{o}}x_{\text{5}}\\ {\dot{x}}_{\text{3}}=x_{\text{4}}\\ {\dot{x}}_{\text{4}}=\frac{k_{c}}{J_{b}}x_{\text{1}}+\frac{c_{c}}{J_{b}}x_{\text{2}}-\frac{k_{b}+k_{c}}{J_{b}}x_{\text{3}}-\frac{c_{b}+c_{c}}{J_{b}}x_{\text{4}}\\ {\dot{x}}_{\text{5}}=-\frac{i_{g}k_{e0}}{L_{m0}}x_{\text{2}}-\frac{R_{m0}}{L_{m0}}x_{\text{5}}+\frac{\text{1}}{L_{m0}}\underset{x_{\text{6}}}{\underbrace{\left(-i_{g}\Delta k_{e}x_{\text{2}}-\Delta R_{m}x_{\text{5}}-\Delta L_{m}{\dot{x}}_{\text{5}}-R_{n}x_{\text{5}}^{\text{3}}+T_{d}\right)}}+\frac{\text{1}}{L_{m0}}u \end{array}\right. \end{array}$$ (22)

A NESO is developed in this paper to estimate the variations of state variables, rather than directly measuring them using physical sensors, thereby reducing operational costs and mitigating the influence of sensor noise. The observation error (*e*_1_) is defined as in equation (23).


$$\begin{array}{c} e_{\text{1}}=x_{\text{5}}-{\hat{x}}_{\text{5}} \end{array}$$ (23)

The structure of the proposed NESO is described by equation (24), where *β*_*i*_ denote the observer gains. The NESO is adopted in this study because the lumped disturbance contains the combined effects of nonlinearities, parametric uncertainties, and external disturbances. Compared with a conventional LESO, the nonlinear error-feedback mechanism provides error-dependent observer correction without relying solely on uniformly high linear observer gains. This characteristic is expected to provide more suitable disturbance estimation for the nonlinear and uncertain electromechanical stabilizer bar dynamics considered in this study, and its effectiveness relative to the LESO is further evaluated numerically in Section 4.


$$\begin{array}{c} \left\{ \begin{array}{c} {\dot{\hat{x}}}_{\text{1}}={\hat{x}}_{\text{2}}+\beta_{\text{1}}f_{\text{1}}\left(e_{\text{1}}\right)\\ {\dot{\hat{x}}}_{\text{2}}=-\frac{k_{c}}{J_{o}}{\hat{x}}_{\text{1}}-\frac{c_{c}+c_{o}}{J_{o}}{\hat{x}}_{\text{2}}+\frac{k_{c}}{J_{o}}{\hat{x}}_{\text{3}}+\frac{c_{c}}{J_{o}}{\hat{x}}_{\text{4}}+\frac{\eta_{g}i_{g}k_{t}}{J_{o}}{\hat{x}}_{\text{5}}+\beta_{\text{2}}f_{\text{2}}\left(e_{\text{1}}\right)\\ {\dot{\hat{x}}}_{\text{3}}={\hat{x}}_{\text{4}}+\beta_{\text{3}}f_{\text{3}}\left(e_{\text{1}}\right)\\ {\dot{\hat{x}}}_{\text{4}}=\frac{k_{c}}{J_{b}}{\hat{x}}_{\text{1}}+\frac{c_{c}}{J_{b}}{\hat{x}}_{\text{2}}-\frac{k_{b}+k_{c}}{J_{b}}{\hat{x}}_{\text{3}}-\frac{c_{b}+c_{c}}{J_{b}}{\hat{x}}_{\text{4}}+\beta_{\text{4}}f_{\text{4}}\left(e_{\text{1}}\right)\\ {\dot{\hat{x}}}_{\text{5}}=-\frac{i_{g}k_{e0}}{L_{m0}}{\hat{x}}_{\text{2}}-\frac{R_{m0}}{L_{m0}}{\hat{x}}_{\text{5}}+\frac{\text{1}}{L_{m0}}{\hat{x}}_{\text{6}}+\frac{\text{1}}{L_{m0}}u+\beta_{\text{5}}f_{\text{5}}\left(e_{\text{1}}\right)\\ {\dot{\hat{x}}}_{\text{6}}=\beta_{\text{6}}f_{\text{6}}\left(e_{\text{1}}\right) \end{array}\right. \end{array}$$ (24)

The nonlinear approximation function (*f*_i_(*e*_1_)) is defined as in equation (25), where *κ* is a positive scaling gain that determines the overall sensitivity of the observer correction term, *ϕ* is a small smoothing constant introduced to avoid singularity when e1 approaches zero, and *λ* is a design parameter that adjusts the nonlinear growth rate of the function. Compared with conventional linear approximation functions, the proposed nonlinear function offers enhanced flexibility and robustness in disturbance estimation. Specifically, it provides a higher correction gain when the observation error is significant, which accelerates the convergence speed of the observer, while yielding a smoother response in the vicinity of the equilibrium point. As a result, excessive amplification of measurement noise and chattering phenomena are effectively suppressed. This nonlinear structure is therefore more suitable for estimating lumped disturbances in the presence of model nonlinearities, parametric uncertainties, and external disturbances.


$$\begin{array}{c} f_{i}\left(e_{\text{1}}\right)=tanh\left[\kappa \frac{e_{\text{1}}}{{\left(\left|e_{\text{1}}\right|+\phi_{i}\right)}^{1-\lambda_{i}}}\right] \end{array}$$ (25)

To ensure the convergence of the proposed NESO, the observer law must satisfy the condition specified in (26), where *k*_1_ denotes a positive constant.


$$\begin{array}{c} {\dot{e}}_{\text{1}}=-k_{\text{1}}f_{\text{1}}\left(e_{\text{1}}\right) \end{array}$$ (26)

**Assumption 1.** The lumped disturbance represented by the augmented state *x*_6_, which includes the effects of external disturbances, model nonlinearities, and parametric uncertainties, is bounded and sufficiently smooth, and its rate of variation is bounded. Moreover, the observer gains *β*_*i*_ are selected such that the internal error dynamics of the NESO are stable. Under these conditions, the state-estimation errors remain bounded, and the augmented-state estimation error converges to a sufficiently small neighborhood of the origin after the observer transient.

**Assumption 2.** After the initial observer transient, the residual lumped-disturbance estimation error is sufficiently small relative to the nominal disturbance-compensated control dynamics. Accordingly, this residual estimation error is neglected in the nominal Lyapunov stability analysis, while its influence under time-varying disturbances and parameter uncertainties is evaluated numerically in Section 4.

**Remark 1.** Assumptions 1 and 2 define the conditions under which the observer convergence and closed-loop stability analyses are established. Assumption 1 ensures that the lumped disturbance and its variation remain within a range that the NESO can handle, thereby supporting bounded and convergent state estimation. Assumption 2 links the observer performance to the subsequent controller stability analysis by allowing the disturbance-compensated dynamics to be analyzed after the observer transient. These assumptions are introduced for theoretical tractability and do not imply perfect disturbance reconstruction; the influence of the remaining estimation error and non-ideal effects is further examined through numerical simulations.

To further characterize the convergence property of the NESO, consider the observation-error dynamics in (26). A Lyapunov candidate associated with the measurable-state observation error is defined in (27).


$$\begin{array}{c} V_{o}=\frac{\text{1}}{\text{2}}e_{\text{1}}^{\text{2}} \end{array}$$ (27)

Taking its time derivative and using (26) gives


$$\begin{array}{c} {\dot{V}}_{o}=e_{\text{1}}{\dot{e}}_{\text{1}}=-k_{\text{1}}e_{\text{1}}f_{\text{1}}\left(e_{\text{1}}\right) \end{array}$$ (28)

Since *k*_1_ > 0 and the nonlinear correction function in (25) is sign-preserving, i.e., *e*_1_*f*_1_(*e*_1_) > 0 for *e*_1_ ≠ 0, it follows that the time derivative of *V*_*o*_ is negative whenever *e*_1_ ≠ 0. Therefore, the output observation error *e*_1_ asymptotically approaches the origin under the observer dynamics in (26). Together with Assumption 1, this property indicates that the remaining state-estimation errors, including the augmented-state estimation error, remain bounded and converge to a small neighborhood determined by the variation rate of the lumped disturbance and the selected observer gains. Consequently, the NESO provides a sufficiently accurate estimate of the lumped disturbance after the initial observer transient.

The derivative of the reference signal (*x*_5*ref*_) is smoothed using a Tracking Differentiator (TD), which serves as a crucial component of the ADRC framework. The structure of the proposed TD is illustrated in (29), where *ϑ* is a positive design constant that determines the tracking speed and smoothing characteristics of the differentiator.


$$\begin{array}{c} \left\{ \begin{array}{c} {\dot{z}}_{\text{1}}=z_{\text{2}}\\ {\dot{z}}_{\text{2}}=-\vartheta_{\text{1}}\left(z_{\text{1}}-x_{5ref}\right)-\vartheta_{\text{2}}z_{\text{2}} \end{array}\right. \end{array}$$ (29)

The motor current (*x*_5_) is selected as the control output and is denoted by *y*, as given in equation (30).


$$\begin{array}{c} y={\hat{x}}_{\text{5}} \end{array}$$ (30)

Equation (31) is obtained by taking the time derivative of *y*.


$$\begin{array}{c} \dot{y}=\underset{\hat{d}}{\underbrace{-\frac{i_{g}k_{e0}}{L_{m0}}{\hat{x}}_{\text{2}}-\frac{R_{m0}}{L_{m0}}{\hat{x}}_{\text{5}}+\frac{\text{1}}{L_{m0}}{\hat{x}}_{\text{6}}+\beta_{\text{5}}f_{\text{5}}\left(e_{\text{1}}\right)}}+\underset{b}{\underbrace{\frac{\text{1}}{L_{m0}}}}u=\hat{d}+bu \end{array}$$ (31)

The tracking error (*e*_2_) is defined as in equation (32).


$$\begin{array}{c} e_{\text{2}}=z_{\text{1}}-{\hat{x}}_{\text{5}} \end{array}$$ (32)

A sliding surface (*s*) is proposed in equation (33).


$$\begin{array}{c} s=e_{\text{2}} \end{array}$$ (33)

By taking the time derivative of the sliding surface, equation (34) is obtained.


$$\begin{array}{c} \dot{s}=z_{\text{2}}-\hat{d}-bu \end{array}$$ (34)

The integrated control law is selected as in equation (35) to compensate for the total disturbance.


$$\begin{array}{c} u=\frac{u_{st}-\hat{d}}{b} \end{array}$$ (35)

In (35), the estimated lumped disturbance provided by the NESO is incorporated as a feedforward compensation term. According to Assumptions 1 and 2, the residual disturbance-estimation error becomes sufficiently small after the observer transient. Therefore, the subsequent nominal stability analysis is conducted for the disturbance-compensated dynamics under Assumption 2. The effect of the nonzero estimation error under time-varying disturbances and parametric uncertainties is further evaluated through the numerical results in Section 4.

The super-twisting control law (*u*_*st*_) is formulated according to (36), where *k*_2_ and *k*_3_ are positive constants and *k*_*f*_ denotes the fuzzy tuning gain.


$$\begin{array}{c} \left\{ \begin{array}{c} u_{st}=z_{\text{2}}+k_{\text{2}}k_{f}{\left|s\right|}^{\frac{\text{1}}{\text{2}}}\Psi \left(s\right)-w\\ \dot{w}=-k_{\text{3}}\Psi \left(s\right) \end{array}\right. \end{array}$$ (36)

The approximation function (Ψ(*s*)) is defined in equation (37), where *ρ* is a positive constant.


$$\begin{array}{c} \Psi \left(s\right)=\frac{s}{\sqrt{s^{\text{2}}+\rho^{\text{2}}}} \end{array}$$ (37)

By combining equations (34), (35), and (36), equation (38) is obtained.


$$\begin{array}{c} \dot{s}=z_{\text{2}}-\hat{d}-b\frac{u_{st}-\hat{d}}{b}=z_{\text{2}}-\left[z_{\text{2}}+k_{\text{2}}k_{f}{\left|s\right|}^{\frac{\text{1}}{\text{2}}}\Psi \left(s\right)-w\right]=-k_{\text{2}}k_{f}{\left|s\right|}^{\frac{\text{1}}{\text{2}}}\Psi \left(s\right)+w \end{array}$$ (38)

The fuzzy fine-tuning parameter (*k*_*f*_) is added to the control law (36) to improve the system’s adaptability under different operating conditions. An adaptive fuzzy gain-scheduling mechanism is developed based on a continuous-time Takagi–Sugeno fuzzy inference structure. The fuzzy system employs two inputs, denoted by *x*_*f*1_ (roll angle) and *x*_*f*2_ (vertical tire force change), which represent selected vehicle dynamic variables used to modulate the control gain. The first input is partitioned into five linguistic terms, while the second input is partitioned into seven linguistic terms, leading to a total of 35 fuzzy rules. Each fuzzy rule is assigned to one of four output groups indexed by *k* ∈ {1, 2, 3, 4}, according to a predefined rule table.

The membership functions and fuzzy rules are designed to provide adequate coverage of the expected operating ranges while maintaining smooth transitions between adjacent fuzzy regions. For *x*_*f*1_, the five membership functions cover approximately −20–20° and consist of trapezoidal functions at the two outer regions, triangular functions in the intermediate regions, and a Gaussian function around the origin. For *x*_*f*2_, the seven membership functions cover approximately −5000–5000 N, with trapezoidal functions at the outer regions, triangular functions in the intermediate regions, and Gaussian functions around the central operating region. This arrangement provides broad coverage near the limits and finer resolution around the central operating region. The 35 input combinations are assigned to the four consequent groups according to a symmetric rule matrix that accounts for the signs and relative magnitudes of the two inputs. Thus, different combinations of roll response and vertical tire force variation activate distinct groups of consequences, while the overlapping membership functions provide smooth transitions between adjacent operating regions.

The firing strength of the (*i*,*j*)^th^ fuzzy rule is defined as in (39), where *μ*_1,*i*_(⋅) and *μ*_2,𝑗_(⋅) denote the membership functions associated with the *i*^*th*^ linguistic term of *x*_*f*1_ and the *j*^*th*^ linguistic term of *x*_*f*2_, respectively. Since all membership functions are non-negative, the firing strengths satisfy *W*_*ij*_ ≥ 0.


$$\begin{array}{c} W_{ij}=\mu_{1,i}\left(x_{f1}\right)\mu_{2,j}\left(x_{f2}\right) \end{array}$$ (39)

Based on the rule grouping, the normalized fuzzy basis functions are constructed as in (40), where Ω_*k*_ denotes the set of fuzzy rules belonging to the *k*^*th*^ output group, and *ε* > 0 is a small positive constant introduced to avoid singularity. By construction, the fuzzy basis functions satisfy *ϕ*_*k*_ ≥ 0 and Σ*ϕ*_*k*_ = 1, which ensures that the fuzzy inference acts as a convex combination of the consequent parameters.


$$\begin{array}{c} \phi_{k}=\frac{\sum_{\left(i,j\right)\in \Omega_{k}}W_{ij}}{\sum_{i=1}^{\text{5}}\sum_{j=1}^{\text{7}}W_{ij}+\varepsilon } \end{array}$$ (40)

To guarantee the non-negativity of the fuzzy output while avoiding explicit inequality constraints on adaptive parameters, a positivity-preserving reparameterization is introduced. Specifically, the consequent parameters are defined as in (41), where *θ*_*k*_ are unconstrained adaptive variables, this formulation ensures *c*_*k*_ ≥ 0 for all *k*, which is particularly important for gain scheduling applications.


$$\begin{array}{c} \begin{array}{cc} c_{k}=\theta_{k}^{\text{2}}, & k=1,\text{\textbackslash{}ldots},4 \end{array} \end{array}$$ (41)

The fuzzy output is computed according to (42), where *α* > 0 is a scaling factor. Owing to the non-negativity of both the fuzzy basis functions and the consequent parameters, the output is guaranteed to be non-negative. It is therefore suitable for modulating the control gain in the proposed finite-time sliding mode control framework.


$$\begin{array}{c} y=\alpha \sum_{k=1}^{\text{4}}\phi_{k}c_{k}=\alpha \phi_{k}^{T}c_{k} \end{array}$$ (42)

The consequent parameters are adjusted online rather than prescribed as fixed values during controller operation. Their adaptive law is defined in (43), where *γ*_*k*_ > 0 denotes the adaptation gain, *e* = *s* is the adaptation signal, and *k*_*d*_ > 0 is the damping coefficient. In the present implementation, the adaptation gains are set to *γ*_*k*_ = 10 for all four consecutive groups, while *k*_*d*_ = 6. The normalized activation level *ϕ*_*k*_ determines the contribution of each fuzzy rule group to the parameter adaptation, whereas *γ*_*k*_ governs the adaptation rate. The damping term limits excessive parameter drift, while the positivity-preserving relation in (41) ensures non-negative consequent parameters.


$$\begin{array}{c} \begin{array}{cc} {\dot{\theta }}_{k}=-\gamma_{k}\phi_{k}e-k_{d}\theta_{k}, & k=1,\text{\textbackslash{}ldots},4 \end{array} \end{array}$$ (43)

The above adaptive fuzzy structure provides a smooth, non-negative, and bounded gain scheduling mechanism that can be directly incorporated into a Lyapunov-based stability analysis. The detailed stability proof of the closed-loop system will be presented in the subsequent subsection.

The output of the adaptive fuzzy system is further employed to construct the gain scheduling factor. Specifically, the scheduling gain is defined as in (44), where *k*_*f,max*_ > 0 denotes the maximum allowable gain modulation coefficient. Since the fuzzy output (*y*) is guaranteed to be non-negative, the scheduling gain satisfies *k*_*f*_ ≥ 1 by construction. This formulation ensures that the baseline control gain is preserved while allowing adaptive gain amplification when required.


$$\begin{array}{c} k_{f}=1+yk_{f,max} \end{array}$$ (44)

**Remark 2.** The STSMC, NADRC, and adaptive fuzzy techniques employed in this study are established control concepts; therefore, the contribution of the proposed framework does not arise from these individual techniques alone, but from their coordinated integration for the coupled electromechanical stabilizer bar–vehicle system. Specifically, the NADRC component employs the NESO to estimate and compensate for the lumped effects of external disturbances, model nonlinearities, and parameter uncertainties. The STSMC component provides robust motor-current tracking in the presence of residual uncertainties while alleviating the chattering associated with conventional first-order SMC. Meanwhile, the adaptive fuzzy mechanism adjusts the control gain online based on the vehicle roll angle and vertical tire force variation, allowing the control action to respond to changes in vehicle dynamic conditions rather than relying solely on fixed control parameters. Thus, rather than addressing disturbance estimation, robust tracking, and gain adaptation as separate control functions, the proposed framework coordinates these complementary functions within the coupled actuator–vehicle dynamics. This integration enables the simultaneous incorporation of disturbance compensation, robust actuator tracking, and operating-condition-dependent gain adaptation into the electromechanical stabilizer bar control framework, which constitutes the main methodological contribution of the proposed approach.

### 3.1 Stability proofs

A Lyapunov candidate function is selected as in equation (45), where *V*_1_ represents the control law, and *V*_2_ represents the fuzzy adaptation law.


$$\begin{array}{c} V=V_{\text{1}}\left(e_{\text{1}},s,w\right)+V_{\text{2}}\left(\theta_{k}\right) \end{array}$$ (45)

As defined in equation (46), the first Lyapunov function (*V*_1_) is positive definite provided that *k*_3_ is a positive constant.


$$\begin{array}{c} \left\{ \begin{array}{c} V_{\text{1}}\left(e_{\text{1}},s,w\right)=\begin{array}{cc} \frac{\text{1}}{\text{2}}e_{\text{1}}^{\text{2}}+\frac{\text{1}}{\text{2}}w^{\text{2}}+k_{\text{3}}\Omega \left(s\right)>0, & \forall \left(e_{\text{1}},s,w\right)\neq \left(0,0,0\right) \end{array}\\ \begin{array}{cc} \Omega \left(s\right)=\underset{\text{0}}{\int^{s}}\Psi \left(\varphi \right)d\varphi =\underset{\text{0}}{\int^{s}}\frac{\varphi }{\sqrt{\varphi^{\text{2}}+\varepsilon^{\text{2}}}}d\varphi =\sqrt{s^{\text{2}}+\varepsilon^{\text{2}}}-\varepsilon >0, & \forall s\neq 0 \end{array} \end{array}\right. \end{array}$$ (46)

According to (47), *V*_2_ is also positive definite since *γ*_*k*_ is positive.


$$\begin{array}{c} \begin{array}{cc} V_{\text{2}}\left(\theta_{k}\right)=\theta_{k}^{T}\gamma_{k}^{-1}\theta_{k}>0, & \forall \end{array}\theta_{k}\neq 0 \end{array}$$ (47)

Under Assumptions 1 and 2, the NESO estimation errors remain bounded, and the residual disturbance-estimation error is sufficiently small after the observer transient. Accordingly, the following Lyapunov analysis is conducted for the nominal disturbance-compensated closed-loop dynamics. By differentiating *V*_1_ and combining it with equations (26), (36), (38), and (46), equation (48) is obtained. Since the time derivative of *V*_1_ is negative definite under the above assumptions, the nominal disturbance-compensated control dynamics associated with the proposed control law are Lyapunov stable.


$$\begin{array}{c} \begin{array}{c} {\dot{V}}_{\text{1}}\left(e_{\text{1}},s,w\right)=e_{\text{1}}{\dot{e}}_{\text{1}}+w\dot{w}+k_{\text{3}}\dot{\Omega }\left(s\right)\\ =-k_{\text{1}}e_{\text{1}}\frac{e_{\text{1}}}{{\left(\left|e_{\text{1}}\right|+\lambda_{i}\right)}^{1-\gamma_{i}}}+w\left[-k_{\text{3}}\Psi \left(s\right)\right]+k_{\text{3}}\Psi \left(s\right)\left[-k_{\text{2}}k_{f}{\left|s\right|}^{\frac{\text{1}}{\text{2}}}\Psi \left(s\right)+w\right]\\ =-k_{\text{1}}\frac{e_{\text{1}}^{\text{2}}}{{\left(\left|e_{\text{1}}\right|+\lambda_{i}\right)}^{1-\gamma_{i}}}\begin{array}{cc} -k_{\text{2}}k_{\text{3}}k_{f}{\left|s\right|}^{\frac{\text{1}}{\text{2}}}\Psi^{\text{2}}\left(s\right)<0, & \forall \left(e_{\text{1}},s,w\right)\neq \left(0,0,0\right) \end{array} \end{array} \end{array}$$ (48)

This result also clarifies the influence of the NESO on the closed-loop stability. The observer reduces the lumped disturbance entering the controlled dynamics to a small residual estimation error after its transient response, thereby allowing the STSMC component to operate on the disturbance-compensated system considered in (48). Hence, the reliability of the closed-loop stability result depends on the boundedness and convergence properties of the NESO stated in Assumption 1, together with the nominal-analysis condition stated in Assumption 2.

Equation (49) is obtained by taking the time derivative of *V*_2_ and combining it with equation (43).


$$\begin{array}{c} {\dot{V}}_{\text{2}}\left(\theta_{k}\right)=\theta_{k}^{T}\gamma_{k}^{-1}\left(-\gamma_{k}\phi_{k}e-k_{d}\theta_{k}\right)=-e\phi_{k}\theta_{k}^{T}-k_{d}\theta_{k}^{T}\gamma_{k}^{-1}\theta_{k}\leq -k_{d}\theta_{k}^{T}\gamma_{k}^{-1}\theta_{k}+\left|e\right|\cdot \|\theta_{k}\| \end{array}$$ (49)

According to the Rayleigh quotient, the inequality in (50) holds.


$$\begin{array}{c} \theta_{k}^{T}\gamma_{k}^{-1}\theta_{k}\geq \lambda_{min}\gamma_{k}^{-1}{\|\theta_{k}\|}^{\text{2}} \end{array}$$ (50)

By combining (49) and (50), the result in (51) is obtained.


$$\begin{array}{c} {\dot{V}}_{\text{2}}\left(\theta_{k}\right)\leq -k_{d}\lambda_{min}\gamma_{k}^{-1}{\|\theta_{k}\|}^{\text{2}}+\left|e\right|\cdot \|\theta_{k}\| \end{array}$$ (51)

By applying Young’s inequality, the inequality in (52) is obtained.


$$\begin{array}{c} \left|e\right|\cdot \|\theta_{k}\|\leq \frac{\text{1}}{\text{2}}{\left|e\right|}^{\text{2}}+\frac{\text{1}}{\text{2}}{\|\theta_{k}\|}^{\text{2}} \end{array}$$ (52)

Substituting (52) into (51) yields (53).


$$\begin{array}{c} {\dot{V}}_{\text{2}}\left(\theta_{k}\right)\leq -\left[k_{d}\lambda_{min}\gamma_{k}^{-1}-\frac{\text{1}}{\text{2}}\right]{\|\theta_{k}\|}^{\text{2}}+\frac{\text{1}}{\text{2}}{\left|e\right|}^{\text{2}}\leq -\Lambda \left(\|\theta_{k}\|\right)+\Gamma \left(\left|e\right|\right) \end{array}$$ (53)

According to condition (54), the fuzzy adaptation law associated with *V*_2_ is input-to-state stable.


$$\begin{array}{c} k_{d}>\frac{\text{1}}{2\lambda_{min}\left(\gamma^{-1}\right)} \end{array}$$ (54)

## 4 Simulation and discussion

A numerical simulation study is conducted to validate the performance of the proposed control scheme. The physical parameters in Table 1 are selected to represent a realistic passenger-vehicle and electromechanical stabilizer bar system. The parameter selection considers physical plausibility, consistency among the vehicle, suspension, tire, and actuator subsystems, and suitability for evaluating the coupled vehicle–actuator dynamics under the investigated steering maneuvers.

**Table 1:** Vehicle specifications.

Symbol	Unit	Value	Symbol	Unit	Value	Symbol	Unit	Value
*C*_11_ *C*_12_	N ⋅ s/m	3120	*K*_11_ *K*_12_	N/m	33100	*K*_*t*11_ *K*_*t*12_	N/m	168000
*C*_21_ *C*_22_	N ⋅ s/m	3070	*K*_21_ *K*_22_	N/m	32500	*K*_*t2*1_ *K*_*t2*2_	N/m	168000
*i*_*g*_	–	42	*k*_*c*_	N ⋅ m/rad	20000	*L*_*m*0_	H	0.004
*R*_*m*0_	Ω	0.44	*k*_*e*0_	V ⋅ s/rad	0.83	*m*_*s*_	kg	1710
*d*_1_	m	1.265	*t*_*w*1_	m	0.775	*η*	–	0.95
*d*_2_	m	1.695	*t*_*w*2_	m	0.775	*c*_*b*_	N ⋅ m ⋅ s/rad	1.8
*J*_*o*_	kg ⋅ m^2^	0.02	*h*_*ϕ*_	m	0.63	*c*_*c*_	N ⋅ m ⋅ s/rad	180
*k*_*b*_	N ⋅ m/rad	5800	*h*_*θ*_	m	0.57	*c*_*o*_	N ⋅ m ⋅ s/rad	0.35
*l*_*b*_	m	0.32	*g*	m/s^2^	9.81	*m*_*u*_	kg	47
*J*_*b*_	kg ⋅ m^2^	0.003	*J*_*θ*_	kg ⋅ m^2^	4250	*k*_*t*_	N ⋅ m/A	0.83
*R*_*n*_	Ω/A^2^	0.001	*J*_*ψ*_	kg ⋅ m^2^	4170	*J*_*ϕ*_	kg ⋅ m^2^	950

Two investigation scenarios are considered (Fig 4a), corresponding to two steering maneuvers: a single lane-change steering maneuver (Case 1) and a J-turn steering maneuver (Case 2). The first case is conducted at a moderate vehicle speed of *v*_1_ = 70 km/h, whereas the second case is evaluated at a significantly higher speed of *v*_2_ = 100 km/h. Model nonlinearities and parametric uncertainties are considered in both scenarios. However, the influence of external disturbances (*T*_*d*_) differs between the two cases, as illustrated in Fig 4b.

**Fig 4:**
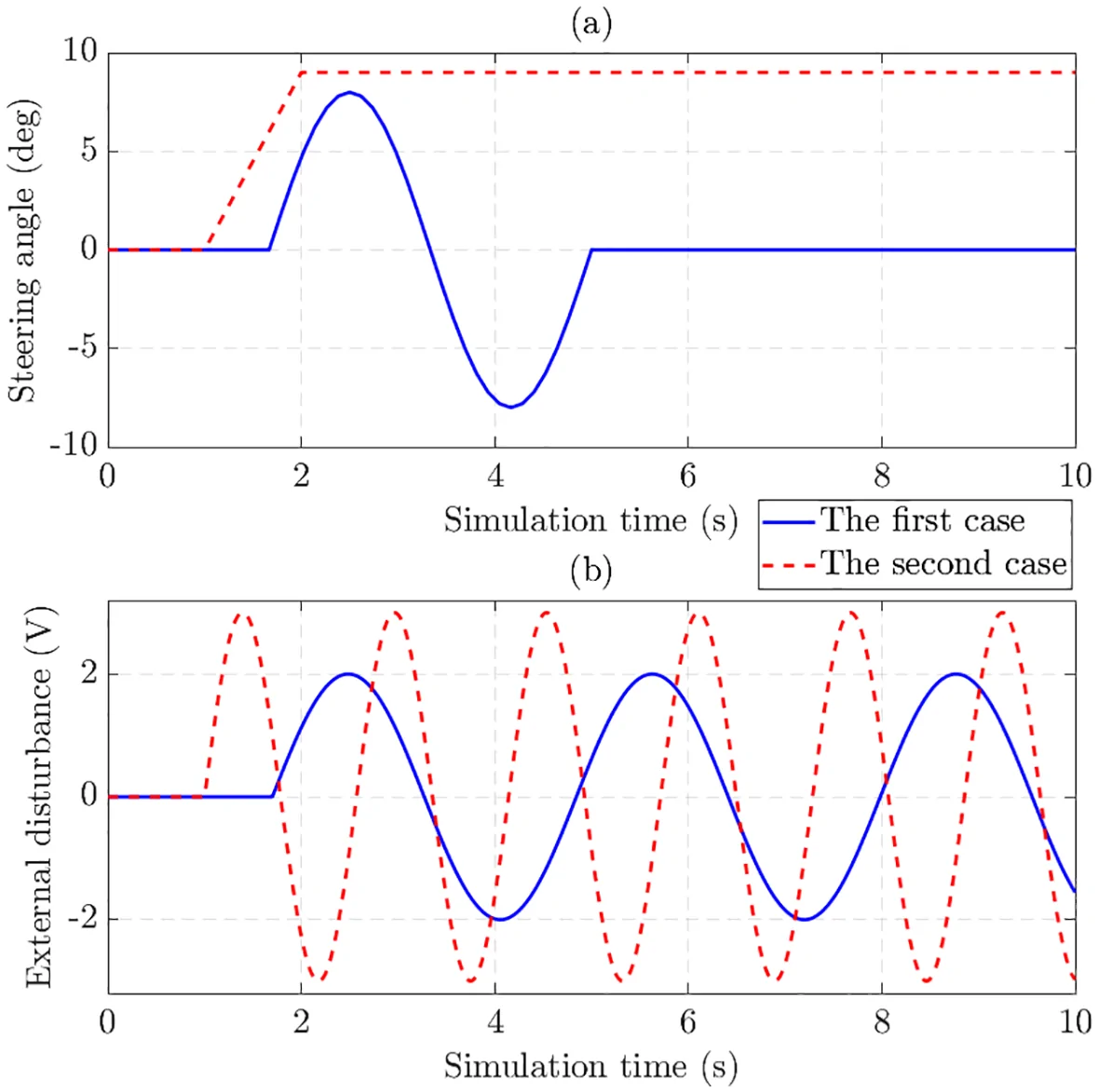
Simulation input. (a) Steering angle. (b) External disturbance.

For each scenario, the results obtained using the proposed control strategy (AFSTSMC) are compared with those achieved by several commonly used controllers, including ADRC, sSMC, and PID. It is worth noting that although the observer gains employed in ADRC are identical to those used in the proposed control, the observer structure and the corresponding control law are substantially different. Additionally, the control law designed for sSMC does not account for the effects of adverse factors.

### 4.1 Single lane change steering

A single lane change steering maneuver is performed at a vehicle speed of *v*_1_ = 70 km/h. The variations in vehicle dynamic behaviors are illustrated by the subplots in Fig 5. As shown in Fig 5a, the roll angle increases with the steering angle and reaches peak values of 6.37° (AFSTSMC), 6.77° (sSMC), 6.56° (ADRC), and 7.05° (PID). Subsequently, these values gradually decrease and return to zero once the steering maneuver is completed. The simulation results indicate that the maximum yaw rate achieved by the proposed control (AFSTSMC) is comparable to that obtained using ADRC, both reaching a value of 0.35 rad/s. In contrast, higher yaw rate peaks are observed with sSMC and PID, reaching 0.37 rad/s and 0.41 rad/s, respectively, as shown in Fig 5b. The variation pattern of the lateral acceleration follows a trend similar to that of the steering input. In this first scenario, the lateral acceleration reaches a maximum of 6.84 m/s^2^ when a PID controls the electromechanical stabilizer bars. This value is reduced to 6.51 m/s^2^ and 6.44 m/s^2^ when ADRC and sSMC are employed, respectively. A slight further improvement is achieved with the proposed control, which limits the lateral acceleration to 6.43 m/s^2^, as illustrated in Fig 5c. Overall, the vehicle trajectories obtained using the proposed control are very close to those achieved with ADRC and sSMC. In contrast, the PID controller exhibits a more noticeable deviation, as shown in Fig 5d.

**Fig 5:**
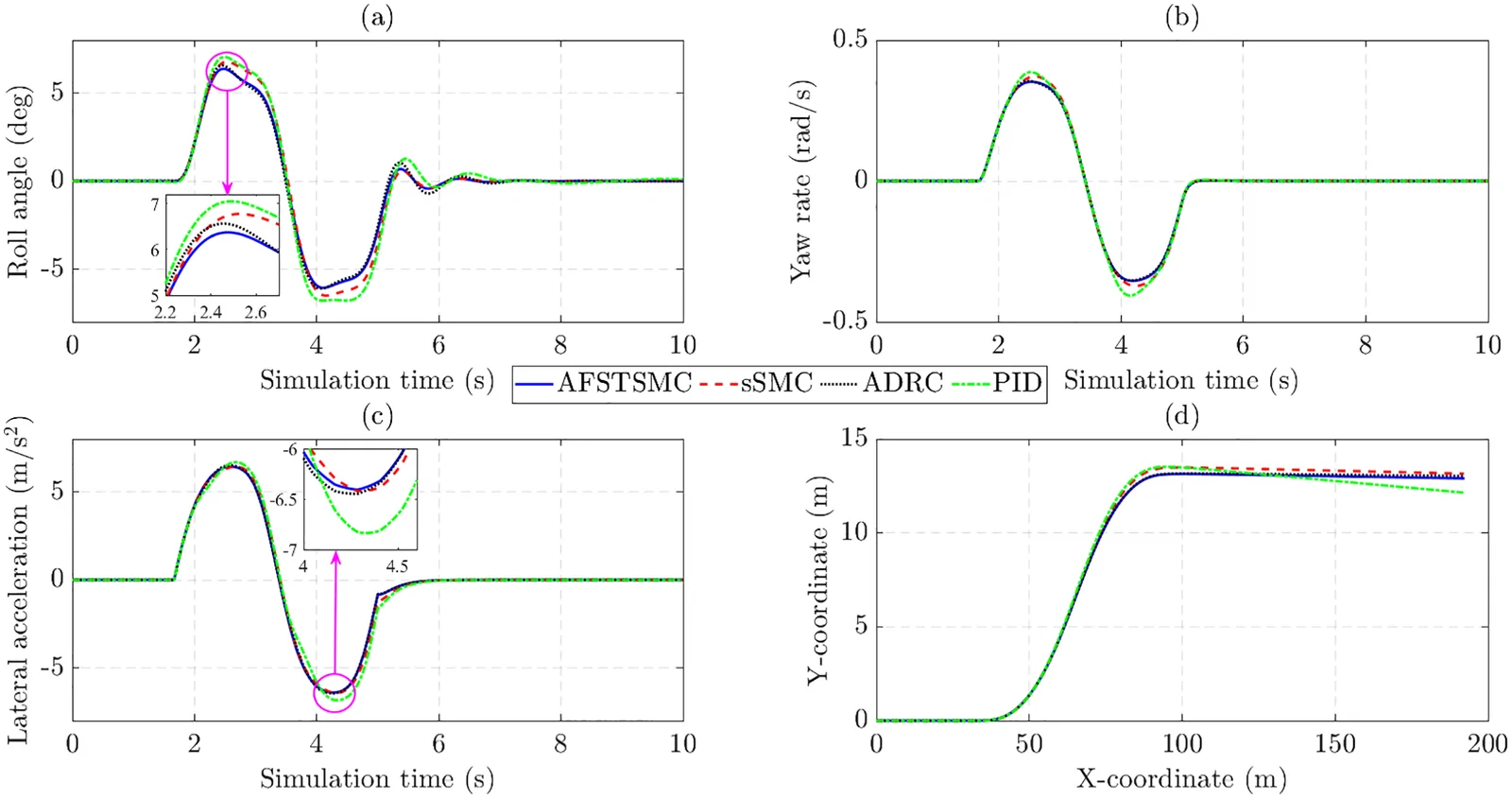
Dynamic behaviors with single lane change steering. (a) Roll angle (b) Yaw rate (c) Lateral acceleration (d) Vehicle trajectory.

Variations in vehicle dynamic behaviors lead to corresponding changes in the vertical tire forces. As shown in Fig 6a, the variation in vertical force remains relatively small during the single lane change maneuver at a vehicle speed of *v*_1_ = 70 km/h. More specifically, the rear tire vertical force (*F*_*z*22_) decreases slightly to 3206.68 N under the proposed control, which is significantly higher than the values obtained using sSMC (2587.34 N), ADRC (2508.23 N), and PID (975.14 N). A closer inspection of Fig 6b and 6d reveals that both the sSMC and PID controllers are significantly affected by adverse factors, including model nonlinearities, parametric uncertainties, and external disturbances, resulting in a loss of convergence capability. Although ADRC can estimate and compensate for these adverse effects by observing the total disturbance, its control law lacks sufficient robustness, resulting in a degradation in overall system performance, as illustrated in Fig 6b.

**Fig 6:**
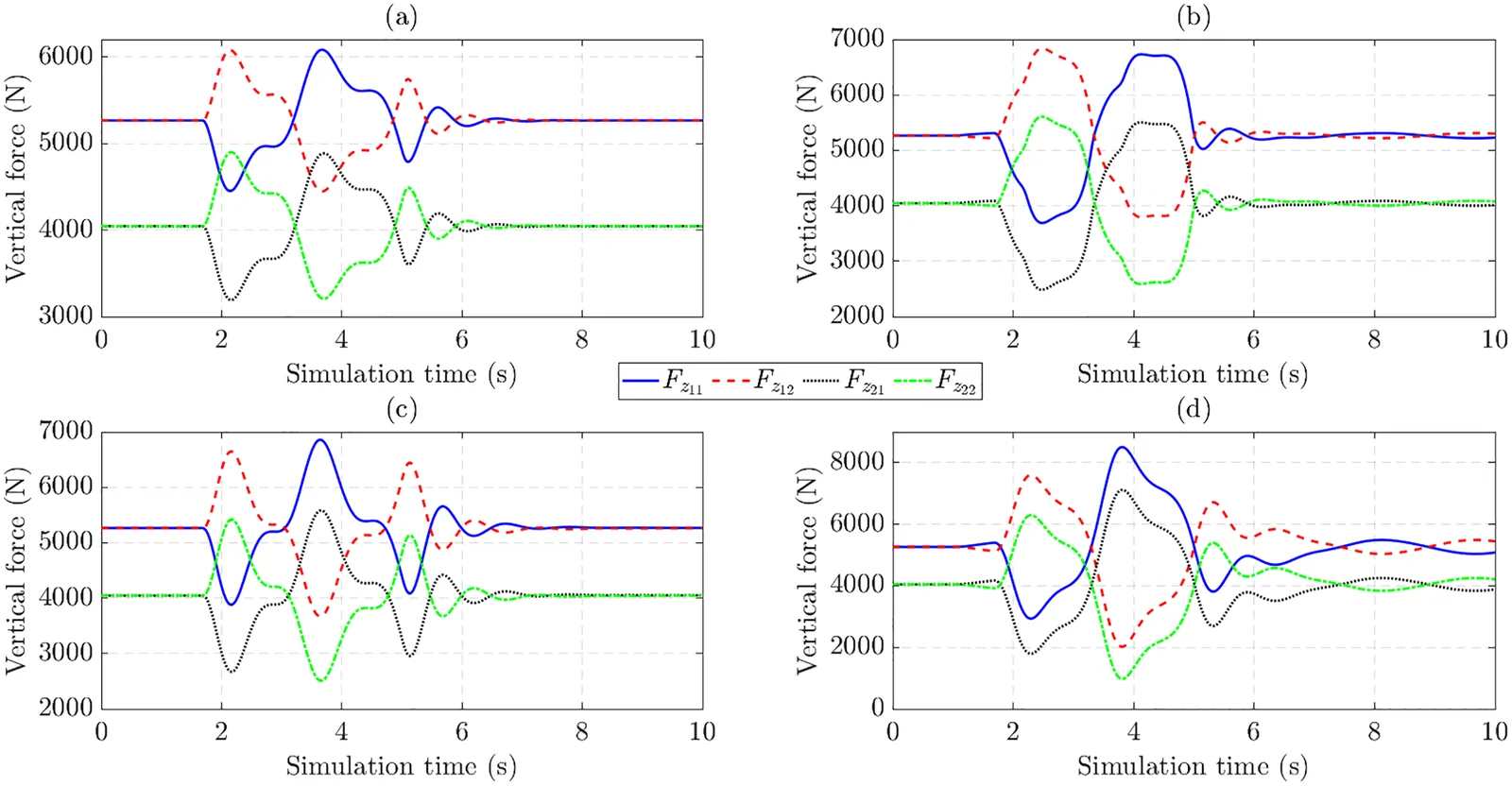
Vertical force with single lane change steering. (a) AFSTSMC (b) sSMC (c) ADRC (d) PID.

The control performance is evaluated in terms of the motor current tracking capability and the actuator force. The simulation results in Fig 7a show that the motor current obtained using AFSTSMC closely follows the reference signal, with a maximum tracking error of only 0.16 A and a Root Mean Square (RMS) error of less than 0.05 A. Under the influence of adverse factors, the maximum error and RMS error associated with sSMC increase dramatically to 10.97 A and 4.12 A, respectively. Although ADRC is capable of compensating for total disturbances, the resulting tracking errors remain relatively large, with a maximum error of 7.26 A and an RMS error of 2.35 A. Moreover, a noticeable phase lag is observed in the ADRC response. In this scenario, the PID controller exhibits the largest tracking error, indicating a significant degradation in performance when the system is subjected to external disturbances, parametric uncertainties, and model nonlinearities. The computed results further show that the actuator force generated by the proposed control closely tracks the desired value, with a maximum error of less than 16.91 N, as illustrated in Fig 7b. In contrast, substantially larger errors are seen with the other controllers, namely 1134.84 N for sSMC, 752.34 N for ADRC, and 2541.60 N for PID.

**Fig 7:**
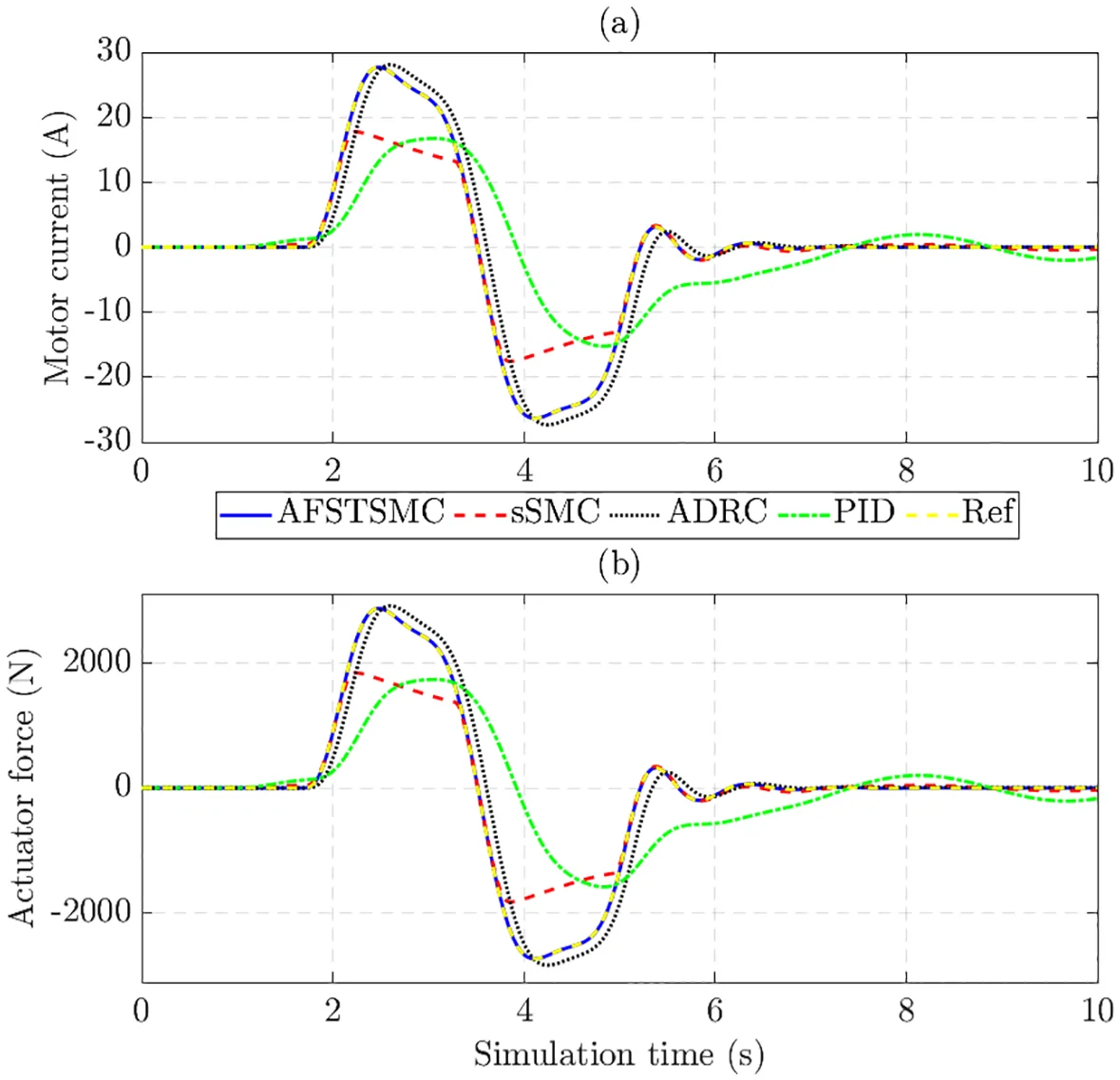
Actuator performance with single lane change steering. (a) Motor current (b) Actuator force.

The observation errors of the NESO (a component of the proposed control) and the LESO (a component of ADRC) are compared in Fig 8. Overall, both NESO and LESO exhibit satisfactory disturbance observation capability in the presence of adverse factors, including parametric uncertainties, external disturbances, and model nonlinearities. However, the quantitative results indicate that NESO provides superior observation performance, yielding minor estimation errors compared to the conventional LESO. More specifically, the RMS error of the augmented state obtained using NESO is 0.088 V, which is slightly lower than that achieved by LESO (0.095 V).

**Fig 8:**
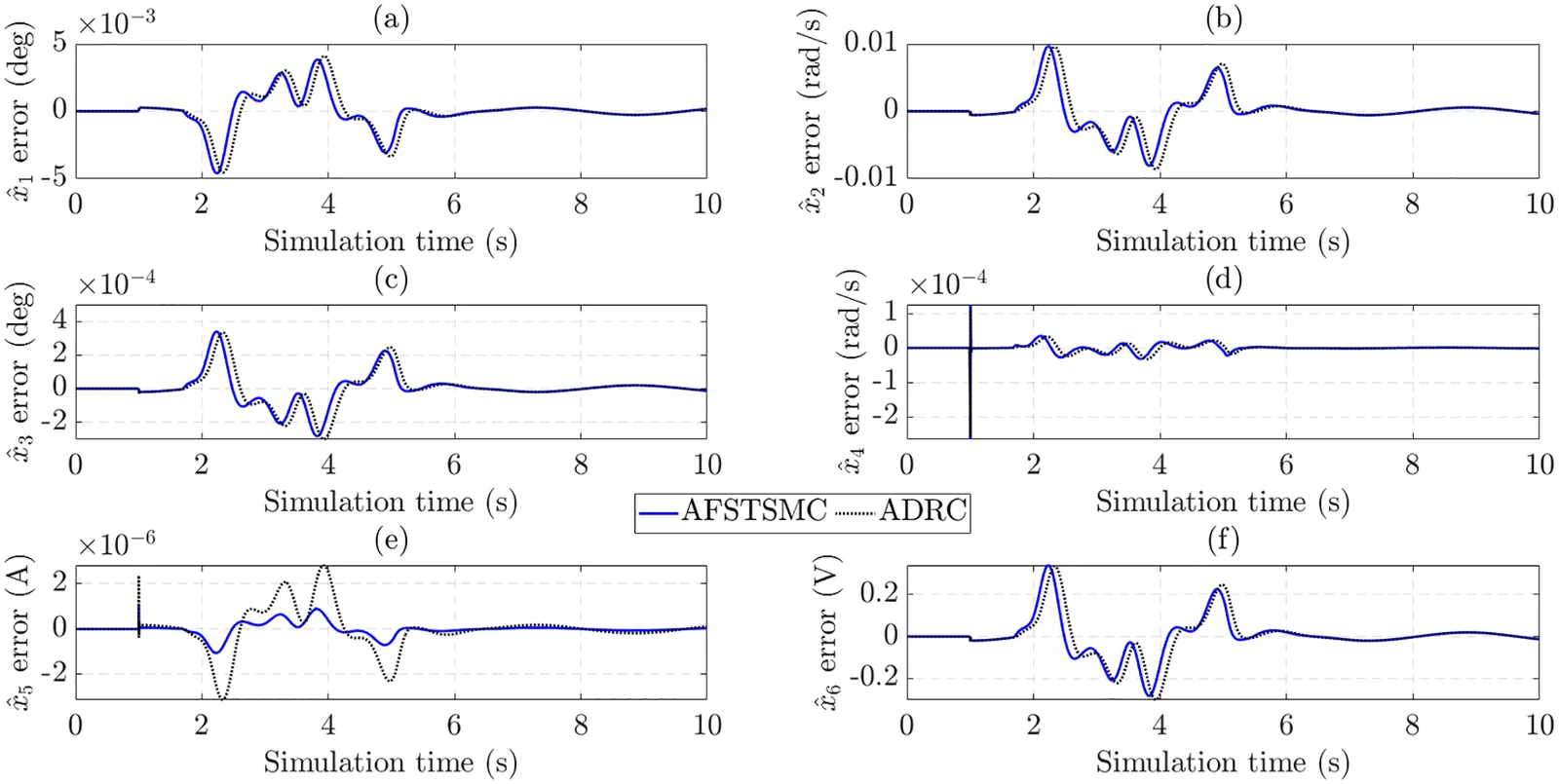
Observer errors with single lane change steering. (a) *x*_1_ error (b) *x*_2_ error (c) *x*_*3*_ error (d) *x*_4_ error (e) *x*_5_ error (f) *x*_6_ error.

### 4.2 J-turn steering

In the second scenario, a high-speed J-turn steering maneuver at *v*_2_ = 100 km/h is investigated. The simulation results in Fig 9a show that the roll angle increases rapidly to a peak value and then oscillates within a small range under the influence of adverse factors. According to the obtained results, the peak roll angles are 7.12° (AFSTSMC), 8.05° (sSMC), 7.19° (ADRC), and 7.80° (PID). Under the effective control of AFSTSMC and ADRC, the yaw rate stabilizes shortly after reaching its peak. In contrast, persistent oscillations are observed in the responses obtained using PID and sSMC, as shown in Fig 9b. In this scenario, the lateral acceleration is minimized when the electromechanical stabilizer bars are controlled by the proposed strategy, reaching a value of 7.74 m/s^2^. In contrast, significantly higher values are obtained with the other controllers, as illustrated in Fig 9c. A closer inspection of Fig 9d further reveals that the turning radius of the vehicle under the proposed control and ADRC is comparable. However, the turning radius achieved with sSMC is considerably smaller, raising concerns regarding potential oversteering behavior.

**Fig 9:**
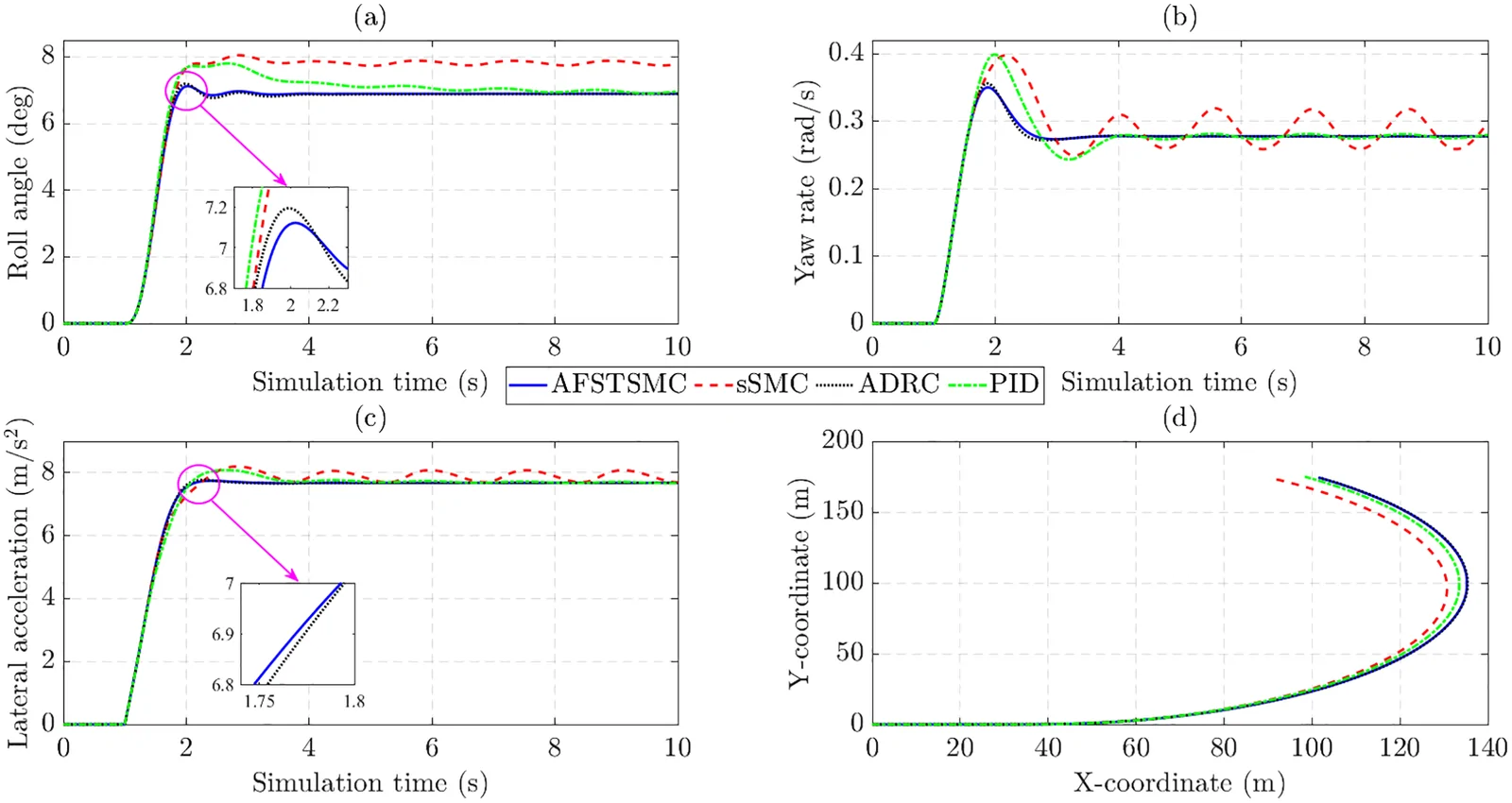
Dynamic behaviors with J-turn steering. (a) Roll angle (b) Yaw rate (c) Lateral acceleration (d) Vehicle trajectory.

Under the influence of adverse factors, the vertical tire forces obtained using sSMC (Fig 10b) and PID (Fig 10d) exhibit persistent oscillations and poor convergence, even when the steering angle is held constant. The simulation results further indicate that the rear tire vertical force (*F*_*z*21_) drops sharply to 1660.01 N with sSMC and 1815.61 N with PID, which may lead to a potential loss of lateral stability under more severe steering conditions. In contrast, AFSTSMC continues to demonstrate superior capability in mitigating the effects of adverse factors, significantly improving the vertical tire force to 3203.33 N, as shown in Fig 10a. This value is considerably higher than that achieved using ADRC, which reaches 2733.06 N, as illustrated in Fig 10c.

**Fig 10:**
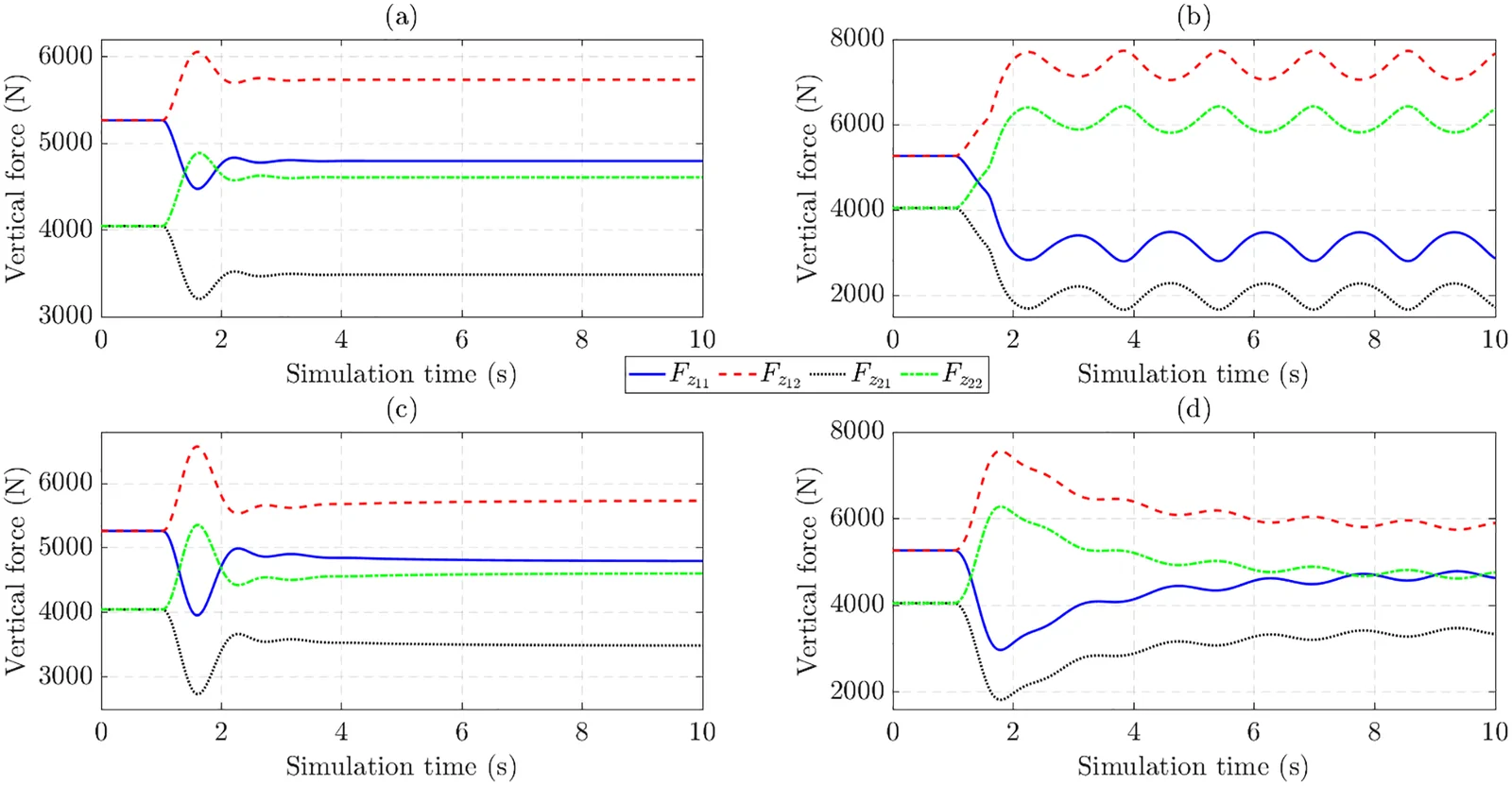
Vertical force with J-turn steering. (a) AFSTSMC (b) sSMC (c) ADRC (d) PID.

The motor current tracking error is minimized when the proposed strategy controls the electromechanical stabilizer bars. As shown in Fig 11a, the maximum tracking error does not exceed 0.11 A, while the RMS error is only 0.02 A, which is significantly lower than the values obtained using ADRC (4.95 A for the maximum error and 1.07 A for the RMS error). In addition, the overshoot in the motor current generated by the proposed control is limited to 3.59%, whereas the corresponding value for ADRC reaches 6.25%. Considering a 2% error band, the time to band and the settling time achieved by the proposed control are 0.84 s (measured from the onset of steering) and 0.17 s (measured after the steering maneuver ends), respectively. In contrast, ADRC yields larger values of 0.92 s for the time to band and 1.38 s for the settling time. The presence of adverse factors leads to a further increase in tracking errors when sSMC and PID control the actuator.

**Fig 11:**
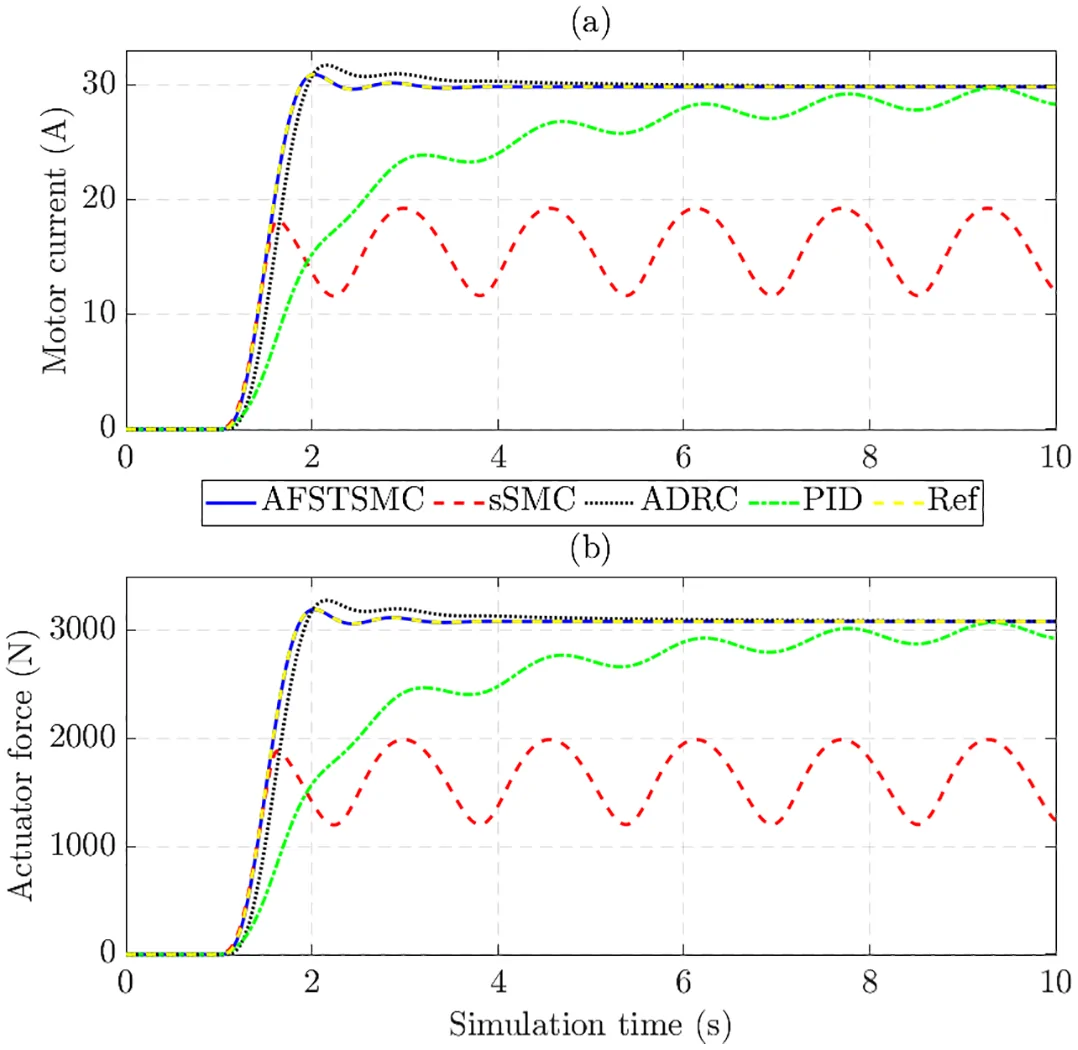
Actuator performance with J-turn steering. (a) Motor current (b) Actuator force.

From the perspective of rollover-risk mitigation, the reductions in vehicle roll response and vertical tire-force variation have direct physical significance. During aggressive steering, lateral acceleration induces lateral load transfer from the inner to the outer wheels, leading to an increasing imbalance in the vertical tire forces and potentially to wheel unloading as the maneuver becomes more severe. The proposed controller suppresses vehicle roll motion by regulating the electromechanical stabilizer bar actuators, and the adaptive fuzzy mechanism further incorporates roll-angle and vertical tire-force variations to adjust the control action according to the instantaneous vehicle condition. Therefore, the reduced roll response and more favorable vertical tire-force distribution observed in the J-turn indicate a lower tendency toward excessive lateral load transfer and inner-wheel unloading, thereby reducing rollover risk. Nevertheless, since an explicit rollover threshold or wheel-lift criterion is not evaluated in this study, these results should be interpreted as evidence of improved rollover resistance rather than a direct guarantee of rollover prevention.

Fig 11b shows that the actuator force generated by AFSTSMC closely follows the reference value, with a maximum error of 11.95 N and an RMS error of 2.43 N. In contrast, significantly larger deviations are observed with the other controllers.

An investigation of the simulation results in Fig 12 reveals that the observation errors obtained using NESO and LESO are relatively close. Nevertheless, NESO still demonstrates superior performance in estimating the total disturbance through the augmented state. The results indicate that the RMS error achieved by NESO (within the proposed control) is 0.082 V, which is slightly lower than that obtained using LESO (within ADRC), at 0.086 V.

**Fig 12:**
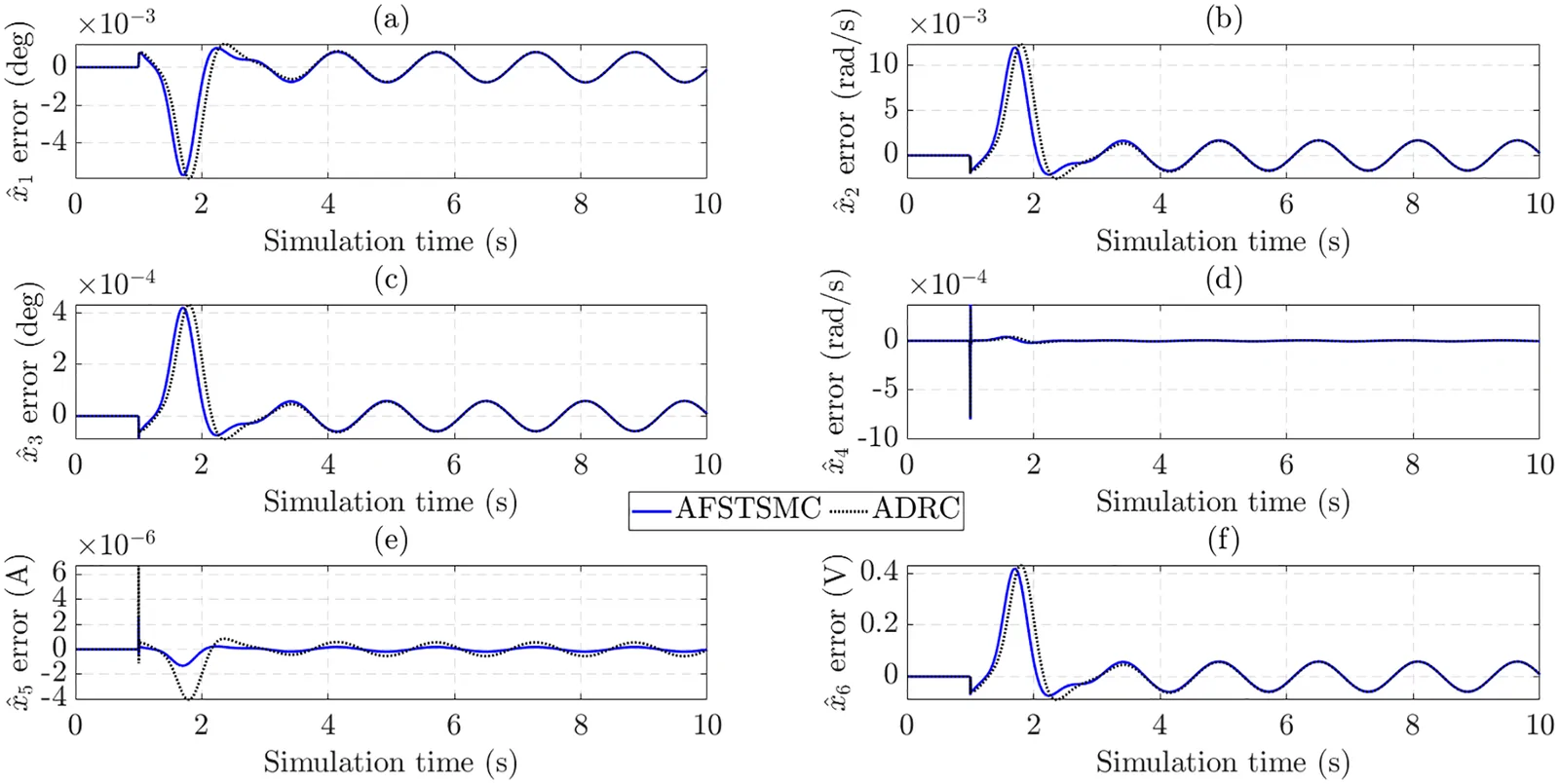
Observer errors with J-turn steering. (a) *x*_1_ error (b) *x*_2_ error (c) *x*_*3*_ error (d) *x*_4_ error (e) *x*_5_ error (f) *x*_6_ error.

### 4.3 Discussion

The simulation results obtained from both scenarios demonstrate that the proposed control strategy delivers superior performance compared with conventional controllers, effectively reducing observation errors, tracking errors, and variations in vertical tire forces. As a result, vehicle dynamic stability is significantly enhanced, thereby mitigating the risk of rollover. In addition, the influence of adverse factors (including parametric uncertainties, model nonlinearities, and external disturbances) is effectively compensated when the proposed approach controls the electromechanical stabilizer bars. Furthermore, chattering and phase lag are completely eliminated, while key transient performance indices (such as overshoot, settling time, and time to band) are significantly reduced.

Accurate current and actuator-force tracking obtained with the coupled model is particularly important because the commanded anti-roll action is not applied directly to the vehicle body. Instead, it is transmitted through the actuator’s electrical and mechanical dynamics before affecting the vehicle’s response. Therefore, the improvements in roll response and vertical tire forces demonstrate the controller’s effectiveness while accounting for non-ideal actuator dynamics, rather than assuming ideal force generation.

Despite the promising performance of the AFSTSMC approach, several limitations remain. First, the parameters of the TD and NESO are selected to ensure system stability but are not strictly optimized. Second, although the output of the fuzzy logic algorithm is adaptively updated, the membership function structure remains fixed, which may limit the achievable performance under a broader range of operating conditions. In addition, the proposed framework requires more online computational effort than conventional PID and ADRC controllers due to the additional NESO, nonlinear STSMC operations, and adaptive fuzzy gain-scheduling mechanism. Nevertheless, the online implementation mainly involves algebraic calculations, observer-state updates, nonlinear functions, and low-dimensional fuzzy inference, without requiring online numerical optimization or iterative solution procedures. Therefore, the computational burden is expected to remain moderate. However, since the present study is limited to numerical simulations, the actual execution time, feasible sampling frequency, and computational delay on automotive embedded hardware have not yet been experimentally quantified. Practical implementation may also be affected by sensor noise, sampling and communication delays, actuator saturation, and uncertainties beyond those considered in the simulations. Under severe operating conditions, these factors may reduce the accuracy of disturbance estimation and degrade tracking and roll-control performance. Finally, the assumptions adopted in the theoretical analysis, together with the simplifications introduced in the numerical model, may lead to discrepancies between the simulation results and actual system behavior. These issues are regarded as existing limitations and will be addressed through real-time implementation and experimental validation in future work.

## 5 Conclusion

This paper presents the design of a robust control strategy aimed at enhancing the performance of active stabilizer bar systems. The proposed control framework is constructed by integrating STSMC with NADRC to effectively compensate for external disturbances, model nonlinearities, and parametric uncertainties, while ensuring system stability and robustness. The control parameters are continuously updated through an adaptive fuzzy gain scheduling mechanism to enhance the system’s adaptability under varying operating conditions.

The numerical results demonstrate that the proposed control approach achieves superior performance compared to the benchmark controllers, resulting in significant reductions in tracking error, overshoot, settling time, and time to band. Moreover, vehicle stability is markedly improved by mitigating variations in vertical tire forces and enhancing overall vehicle dynamic behaviors. Nevertheless, the proposed control method still faces challenges related to optimizing control parameters and refining membership function structures. Future work will focus on experimental validation to further demonstrate the effectiveness and practicality of the proposed approach.
